# Proteomic Signatures of High-Risk Coronary Plaque Features and Incident Events

**DOI:** 10.1016/j.jacbts.2026.101640

**Published:** 2026-07-22

**Authors:** Anish Karpurapu, Lydia Coulter Kwee, Caroline de Calvacamp, Elizabeth Zhao, Kristin Corey, Khurram Nasir, Maros Ferencik, Mette Nyegaard, Peter Loof Møller, Palle Duun Rohde, Hannes Helgason, Morten Bøttcher, Simon Winther, Borek Foldyna, Pamela S. Douglas, Svati H. Shah

**Affiliations:** a Duke University School of Medicine, Durham, North Carolina, USA; b Duke Molecular Physiology Institute, Duke University School of Medicine, Durham, North Carolina, USA; c Department of Medicine, New York-Presbyterian/Columbia University Irving Medical Center, New York, New York, USA; d Houston Methodist DeBakey Heart & Vascular Center, Houston, Texas, USA; e Knight Cardiovascular Institute, Oregon Health and Science University, Portland, Oregon, USA; f Department of Health Science and Technology, Aalborg University, Aalborg, Denmark; g deCODE genetics, Reykjavik, Iceland; h Department of Cardiology, Gødstrup Hospital, Herning, Denmark; i Department of Radiology, Massachusetts General Hospital, Boston, Massachusetts, USA; j Division of Cardiology, Department of Medicine, Duke University School of Medicine, Durham, North Carolina, USA; k Duke Clinical Research Institute, Duke University School of Medicine, Durham, North Carolina, USA

**Keywords:** atherosclerosis, biomarkers, coronary artery disease, coronary computed tomographic angiography, Olink, vulnerable plaque

## Abstract

Patients with and without obstructive coronary artery disease (oCAD) experience heterogeneous cardiovascular risk, and coronary computed tomography angiography (CCTA) high-risk plaque features are not universally available. This study evaluated whether circulating proteomic profiles clarify plaque biology and improve risk assessment. Proteomic profiling of 572 proteins was performed in PROMISE (N = 1,724), with validation in Dan-NICAD (N = 2,743) and UK Biobank (N = 53,018). A high-risk composite phenotype (HRCP) combined oCAD, high stenosis or plaque burden, high coronary calcium, and high-risk plaque features. Thirty-seven proteins were independently associated with HRCP and were mapped to inflammatory, metabolic, and proteolytic pathways; 7 were also associated with major adverse cardiovascular events, including lipoprotein lipase and cathepsin D. While the 37-protein score added little discrimination beyond clinical factors, it improved reclassification (net reclassification index = 0.13, *P* < 0.001), suggesting proteomic profiles may complement clinical models while elucidating disease biology and highlighting cathepsin D as a potential therapeutic target.

## Highlights

• Proteomic profiling identifies protein signatures of high-risk plaque phenotype.
• 37 proteins associate with high-risk plaque; 7 also track cardiovascular events.
• A protein score improves reclassification but adds little discrimination.
• Mendelian randomization flags cathepsin D as a potential causal, druggable CAD target.

## Visual Abstract

**Figure:**
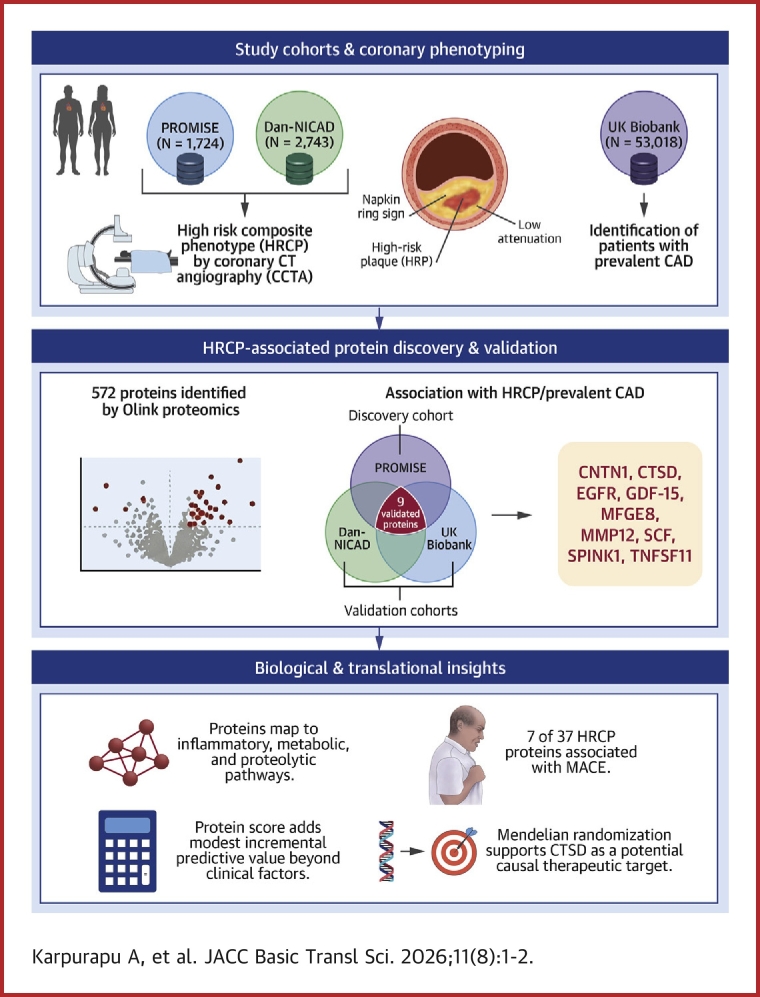

Cardiovascular disease remains a leading cause of morbidity and mortality worldwide, with coronary artery disease (CAD) representing a significant burden on patients and health care systems. Current treatment guidelines base recommendations on the burden of obstructive CAD (oCAD),^1^^,^^2^ but there exists marked heterogeneity in the relationship between CAD severity and cardiovascular event risk. Cardiovascular events occur in patients without oCAD, including in two-thirds of patients with stable angina and over 10% of patients with non–ST-segment elevation acute coronary syndrome.^3^

The heterogeneity of CAD outcomes may be partly explained by differences in plaque characteristics. Certain plaque features, such as thin fibrous caps or high plaque burden, have been linked to an elevated risk of recurrent cardiovascular events.^4^^,^^5^ Noninvasive high-risk plaque (HRP) phenotyping via coronary computed tomography angiography (CCTA) has emerged as a promising tool for risk assessment.^4^, ^5^, ^6^, ^7^, ^8^ The HRP phenotype, marked by traits such as low computed tomography attenuation plaque, positive remodeling, napkin-ring sign, and high noncalcified plaque volume, has shown high prognostic accuracy in predicting cardiac events.^5^^,^^9^, ^10^, ^11^, ^12^

Although HRP findings help explain cardiovascular event risk, the molecular mechanisms driving high-risk plaque formation and rupture remain poorly understood. Recent advancements in molecular omics, including proteomics, genomics, and metabolomics, offer new ways to understand CAD beyond structural characteristics.^13^ for example, adrenomedullin was identified as a novel biomarker of cardiovascular disease risk, particularly heart failure, through proteomic meta-analysis and Mendelian randomization,^14^ and a drug targeting adrenomedullin, adrecizumab, is in clinical trials for heart failure.^15^ While studies like these have offered valuable insights, few have combined comprehensive proteomics with high-resolution imaging of coronary plaque. The integration of molecular insights with artificial intelligence–driven imaging is a rapidly growing field aimed at deepening our understanding of disease mechanisms and progression.^16^, ^17^, ^18^

Here, we performed comprehensive proteomic analysis in patients at low to intermediate risk of CAD to characterize circulating biomarkers beyond established clinical and lipid markers that might report on high-risk coronary plaque biology and incident events. We leveraged PROMISE (PROspective Multicenter Imaging Study for Evaluation) and Dan-NICAD (Danish study of Non-Invasive testing in Coronary Artery Disease), 2 large clinical studies, with a CCTA-guided approach for CAD work-up, to identify molecular pathways related to HRP and CAD.^19^ We identified proteomic biomarkers associated with HRP phenotypes and major adverse cardiovascular events (MACE) and validated these findings in the UK Biobank (UKB).

## Methods

### Study populations

Three independent cohorts were used: PROMISE, Dan-NICAD, and UKB (Table 1, Supplemental Figure 1, Supplemental Tables 1 and 2). The Duke Institutional Review Board approved the analysis, and all participants provided informed consent.

**Table 1:** Baseline Characteristics of the PROMISE Cohort

Characteristic	Overall (N = 1,417)	HRCP (n = 822)	No HRCP (n = 595)	*P* Value
Age, y	60.2 ± 8.1	61.7 ± 8.5	58.0 ± 7.0	<0.001
Self-reported race				0.019^a^
Multiracial	15 (1.1%)	7 (0.9%)	8 (1.3%)	
White	1,243 (87.7%)	738 (89.8%)	505 (84.9%)	
Black or African American	119 (8.4%)	55 (6.7%)	64 (10.8%)	
Asian	22 (1.6%)	13 (1.6%)	9 (1.5%)	
American Indian or Alaska Native	10 (0.7%)	3 (0.4%)	7 (1.2%)	
Native Hawaiian or other Pacific Islander	2 (0.1%)	2 (0.2%)	0 (0%)	
Female sex	726 (51.2%)	329 (40.0%)	397 (66.7%)	<0.001
Diabetes	282 (19.9%)	204 (24.8%)	78 (13.1%)	<0.001
Hypertension	920 (64.9%)	557 (67.8%)	363 (61.0%)	0.010
LDL-C	121 ± 34.7	120 ± 34.5	123 ± 35.1	0.087
BMI (kg/m^2^)	30.5 ± 5.85	30.3 ± 5.51	30.7 ± 6.28	0.26
Statin use	606 (42.8%)	391 (47.6%)	215 (36.1%)	<0.001
Smoker	750 (52.9%)	480 (58.4%)	270 (45.4%)	<0.001
Presence of HRP	267 (18.8%)	267 (32.5%)	0 (0%)	<0.001
Obstructive CAD (≥50%)	238 (16.8%)	238 (29.0%)	0 (0%)	<0.001
Leaman score >5	755 (53.3%)	755 (91.8%)	0 (0%)	<0.001
CAC score >400	204 (14.4%)	204 (24.8%)	0 (0%)	<0.001

Values are presented as mean ± SD or n (%). Unless otherwise indicated, continuous variables were compared using Student's *t*-tests and categorical variables with chi-square tests. HRCP components (HRP, obstructive CAD, CAC >400, Leaman >5) are not mutually exclusive and overlap within individuals. By definition, no HRCP participants have 0% prevalence for all HRCP components.

BMI = body mass index; CAC = coronary artery classification; CAD = coronary artery disease; HRCP = high-risk composite phenotype; HRP = high-risk plaque; LDL-C = low-density lipoprotein cholesterol.

a *P* values from Fisher's exact test.

The PROMISE clinical trial enrolled 10,003 outpatients without any prior diagnosis of CAD who presented with symptoms suspicious for CAD (NCT01174550).^20^ Participants in the study were randomized to one of 2 groups: 1) standard of care or 2) anatomical testing with CCTA. This study included all available participants from the CCTA arm who provided blood biospecimens.

The Dan-NICAD validation cohort consisted of patients from the Dan-NICAD I and II (N = 2,743) multicenter trials in Denmark (NCT02264717, NCT03481712). The trials recruited individuals with symptoms suggestive of CAD but no known CAD.^21^^,^^22^

The UKB is a large, prospective population-based cohort that includes extensive genetic, biochemical, and electronic health record data on ∼500,000 participants across the United Kingdom. This substudy used data from 53,018 participants with proteomic profiling available.

### Outcomes

The primary outcome in PROMISE was a high-risk composite phenotype (HRCP) derived from CCTA reads at a central core laboratory (Massachusetts General Hospital, Boston, MA). HRCP was defined as oCAD (at least 50% stenosis in a major coronary vessel),^4^ high coronary artery calcification (CAC; Agatston score >400),^23^ presence of at least 1 HRP feature, or a high burden of plaque and stenosis (Leaman score >5).^24^ HRP features included napkin-ring sign (higher peripheral attenuation of noncalcified plaque), positive remodeling (remodeling index >1.1), or low attenuation plaque (<30 Hounsfield units).^5^^,^^9^, ^10^, ^11^, ^12^ HRCP controls were defined as individuals without any of these features and without nonobstructive CAD. Each of the HRCP criteria was also considered individually as a secondary outcome. Although PROMISE excluded participants with a prior known CAD diagnosis, given the symptomatic patient population, baseline CCTA could identify previously unrecognized obstructive CAD. Incident MACE in PROMISE was defined as death, incident nonfatal myocardial infarction, or hospitalization for unstable angina.

In Dan-NICAD, imaging outcomes were evaluated by core laboratory personnel (Cardiac Imaging Center, Department of Cardiology, Gødstrup Hospital, Denmark). HRCP cases and controls were defined as in PROMISE.

In UKB, because CCTA imaging is not available, prevalent CAD was used as the primary outcome, defined as CAD that was diagnosed before enrollment, derived from self-report, electronic health record data, and death registry data (Supplemental Methods UK Biobank Outcomes).

### Laboratory Methods

Proteomic profiling in PROMISE was performed on plasma using 7 Olink Target 96 panels (Olink Biosciences): CVD II and III, Development, Inflammation, Metabolism, Oncology III, and Cell Regulation (644 assays). After quality control (Supplemental Methods PROMISE Laboratory Methods), the analysis dataset included log2-transformed normalized protein expression (NPX) values for 572 unique proteins. Proteomic profiling from Dan-NICAD and UKB were derived from the Olink Explore platform,^25^ with assays performed by deCODE genetics and Olink, respectively.^25^

### Statistical analysis

Baseline characteristics are presented using mean ± SD for continuous variables or count (percentage) for categorical variables. HRCP groups were compared using the Student's *t*-test for continuous variables and the Fisher's exact test for categorical variables. In the PROMISE discovery cohort, each of 572 proteins was first tested for association with HRCP using univariable logistic regression models, with results reported as the odds ratio (OR) with 95% CI. False discovery rate (FDR) adjustment using the Benjamini-Hochberg method (*q* < 0.05) was applied for initial protein discovery, with subsequent tests using a nominal *P* < 0.05. Proteins significant in univariable analyses were tested in multivariable analyses (adjusted for age, sex, self-reported race, body mass index, low-density lipoprotein cholesterol (LDL-C), diabetes, hypertension, statin use, and smoking status). This 2-stage approach was chosen to prioritize proteins with direct associations with HRCP, with FDR correction also applied to multivariable results across all 572 proteins as a sensitivity analysis (Supplemental Methods). These significant proteins were also subsequently tested for association with time-to-MACE in PROMISE using Cox proportional hazard models (using the same covariates) reported as the hazard ratio (HR) with 95% CI. Kaplan-Meier curves were used to estimate the yearly MACE incidence rates, and pathway analysis was performed using Gene Set Enrichment Analysis (GSEA).^26^, ^27^, ^28^, ^29^ Two-sample cis-Mendelian randomization was conducted to infer causality using protein quantitative trait loci (pQTLs) identified from a previously published genome-wide association study meta-analysis of CAD, given a lack of genetic studies of HRCP.^30^ Causal estimates were derived using the inverse-variance weighted method for multiple pQTLs and the Wald ratio method for single pQTLs. Phenoclustering was performed using *K*-means clustering to discern groups of patients with similar features, using a large set of clinical and imaging variables (Supplemental Table 4). Analysis of variance (ANOVA) followed by Tukey's post hoc test for multiple pairwise comparisons was used to compare clusters for significant variables (*P* < 0.05). To develop a predictive protein score for HRCP, an elastic net penalized regression model was trained in the PROMISE cohort using the 37 proteins associated with HRCP in PROMISE multivariable models (Supplemental Methods Statistical Analysis).

In Dan-NICAD, validation analyses of the 37 proteins associated with HRCP in PROMISE were performed using analogous univariable and multivariable logistic regression models. The elastic net protein score derived from PROMISE was calculated for Dan-NICAD participants and tested for association with HRCP, along with a model including clinical covariates only and one including clinical covariates plus the protein score. Similar analyses were performed in UKB using prevalent CAD as the outcome instead of HRCP. Additionally, proteins were tested for association with time to incident CAD in UKB using Cox models (Supplemental Methods Statistical Analysis). All analyses were performed in R version 4.1.3.^31^ Elastic net penalized logistic regression was implemented using glmnet with cross-validation via caret.^32^^,^^33^ Receiver operating characteristic curves and areas under the curve (AUCs) were computed using pROC.^34^ Cox proportional hazards models and Kaplan-Meier estimates used the survival package.^35^ Reclassification metrics were calculated using PredictABEL.^36^ Two-sample Mendelian randomization was performed using TwoSampleMR.^37^ Pathway analysis used the *fgsea* package, with KEGG and Hallmark gene sets obtained from MSigDB via *msigdbr*.^26^, ^27^, ^28^, ^29^

## Results

### Baseline characteristics

The mean age in the PROMISE analysis set (N = 1,724) was 60.2 years (SD: 8.0), with 52.8% female participants, and the cohort was predominantly self-reported White race (87.2%) with a high prevalence of cardiovascular risk factors including a mean body mass index of 30.6 (SD: 5.9), 19.9% with diabetes, and 64.4% with hypertension (Supplemental Table 5). Among 1,417 participants with HRCP defined, 822 (58.0%) were HRCP cases, with 18.8% having HRP features, 16.8% having oCAD, 14.4% having CAC >400, and 53.3% having a Leaman score >5. HRCP cases tended to be older than controls with no HRCP (61.7 ± 8.5 vs 58.0 ± 7.0; *P* < 0.001), were less likely to be self-reported Black or African American race (6.7% vs 10.8%; *P* < 0.001), and were more likely to be male (60% vs 33.3%; *P* < 0.001), have diabetes (24.8% vs 13.1%; *P* < 0.001) and smoke (58.4% vs 45.4%; *P* < 0.001) (Table 1). HRCP was also strongly associated with incident MACE (unadjusted HR: 4.20 [95% CI: 1.88-9.37], *P* < 0.001; adjusted HR: 3.22 [95% CI: 1.37-7.55], *P* = 0.007). Kaplan-Meier estimates demonstrated higher cumulative MACE incidence among HRCP participants compared with non-HRCP controls (1 year: 3.10% vs 0.51%; 2 years: 4.48% vs 0.70%; 3 years: 6.81% vs 2.05%).

### Proteins associated with HRCP in PROMISE

**Figure 1:**
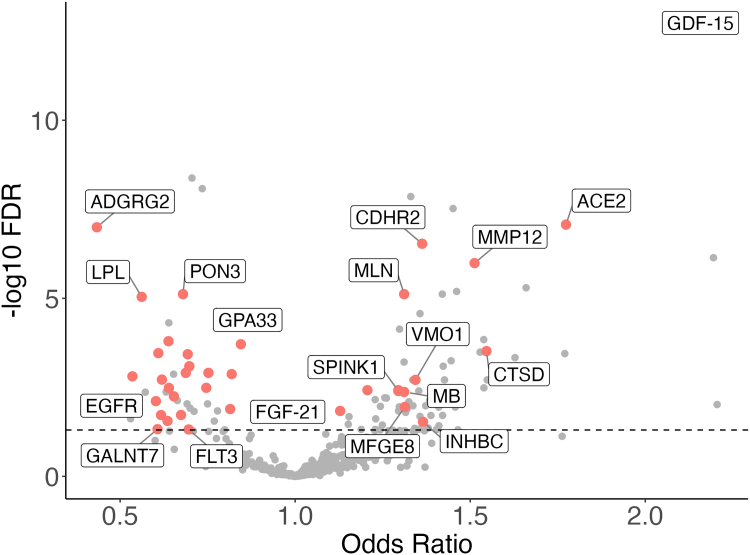
Association of Proteins With HRCP in PROMISE Volcano plot with odds ratios (x-axis) and FDR-adjusted *P*-values (y-axis) derived from univariable logistic regression models for HRCP. The dotted line represents FDR cutoff significance in univariable logistic regression models (*q* < 0.05). Red dots correspond to the proteins that remained significant in multivariable analyses (*P* < 0.05). FDR = false discovery rate; HRCP = high-risk composite phenotype.

In univariable analyses, 114 of 572 proteins tested were associated with HRCP after FDR adjustment for multiple comparisons (*q* < 0.05) (Supplemental Table 6). Of these, 37 proteins remained associated with HRCP in multivariable analyses (*P* < 0.05) (Figure 1, Supplemental Table 7). Here, aOR represents the odds ratio of a 1-NPX increase in protein, corresponding to a doubling of circulating protein level. This group included proteins important in metabolic regulation: GDF-15 (growth differentiation factor–15; aOR: 1.4 [95% CI: 1.1-1.7], *P* = 0.006), FGF21 (fibroblast growth factor–21; aOR: 1.1 [95% CI: 1.0-1.3], *P* = 0.004), LPL (lipoprotein lipase; aOR: 0.7 [95% CI: 0.6-0.9], *P* = 0.013), PON3 (paraoxonase 3; aOR: 0.7 [95% CI: 0.6-0.9], *P* = 0.001); protein degradation (CTSD [cathepsin D]; aOR: 1.3 [95% CI: 1.0-1.6], *P* = 0.045), CTRC (chymotrypsin-C; aOR: 0.8 [95% CI: 0.7-1.0], *P* = 0.027); and inflammation (CCL20 [C-C motif chemokine 20], aOR: 1.2 [95% CI: 1.1-1.4], *P* = 0.002), IL8 (interleukin 8, aOR = 1.2 [95% CI: 1.0-1.4], *P* = 0.015), TNFSF11 (tumor necrosis factor superfamily member–11; aOR: 0.8 [95% CI: 0.6-1.0], *P* = 0.015), CD300LG (CD300 molecule like family member g; aOR: 0.7 [95% CI: 0.5-0.9], *P* = 0.002) and TNFSF12 (tumor necrosis factor superfamily member–12; aOR: 0.6 [95% CI: 0.5-0.8], *P* < 0.001). Among the 4 HRCP subcomponents, Leaman score emerged as the primary driver of protein associations, as multivariable analyses revealed it was linked to a greater number of proteins than the other subcomponents (Supplemental Figures 2 to 5).

To address potential type II error for proteins uncovered only in multivariable models, and to place results in context of the full complement of proteins, in sensitivity analyses, we analyzed multivariable models applying FDR correction across all 572 proteins. These results highlighted 3 proteins remaining significant (*q* < 0.05): BOC (aOR: 0.5 [95% CI: 0.3-0.7], *q* = 0.034), SCF (aOR: 0.6 [95% CI: 0.4-0.8], *q* = 0.034), and ADGRG2 (aOR: 0.5 [95% CI: 0.4-0.8], *q* = 0.045). All 3 were among the original 37 HRCP-associated proteins.

### Association of HRCP proteins with MACE events in PROMISE

**Figure 2:**
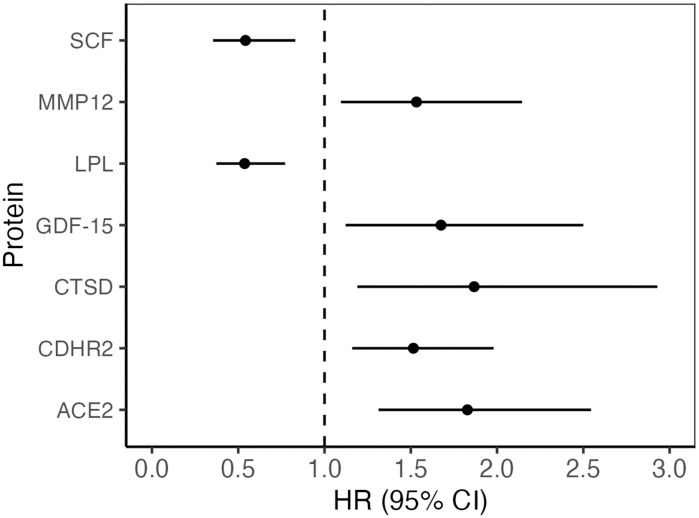
Proteins Significantly Associated With Time to MACE in PROMISE Association of proteins with time to MACE (all-cause death, myocardial infarction, or hospitalization for unstable angina). The plot displays hazard ratios (HRs) from multivariable Cox proportional hazards models adjusted for age, sex, diabetes, hypertension, low-density lipoprotein cholesterol, body mass index, statin use, and smoking history. Hazard ratios represent risk per 1-unit increase in protein values, corresponding to a doubling of circulating protein level. MACE = major adverse cardiovascular events.

**Figure 3:**
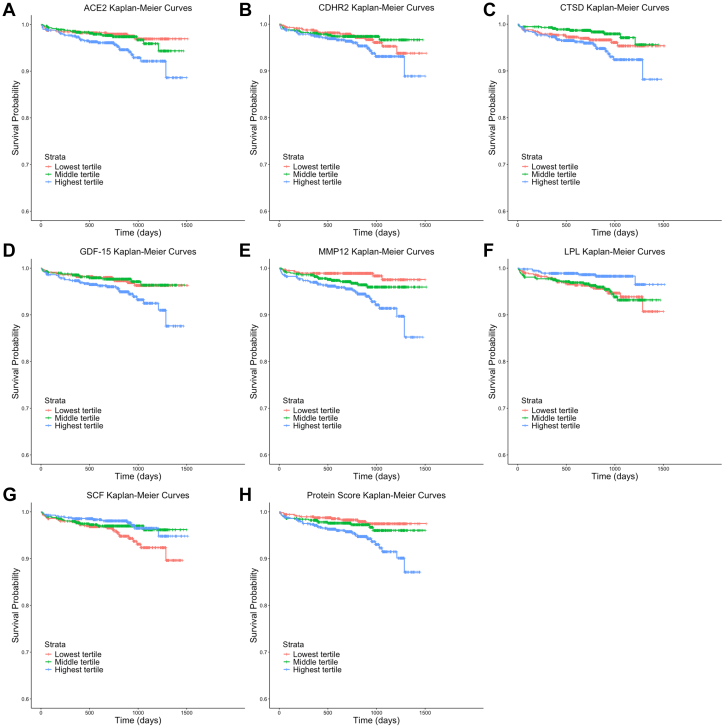
Survival Curves for Individual Proteins and Protein Score Associated With Time to MACE in PROMISE Kaplan-Meier survival curves for MACE in the PROMISE cohort. Survival probability is plotted over time for participants stratified by tertiles of baseline protein or protein score levels. MACE = major adverse cardiovascular events.

In the PROMISE cohort, over a mean follow-up of 25.2 (SD: 10.5) months, a total of 59 individuals experienced a composite MACE event. Of the 37 proteins associated with HRCP, 7 proteins were associated with time-to-event (*q* < 0.05) in univariable models, and all 7 remained significant in multivariable analyses adjusted for clinical risk factors (Figure 2, Figure 3A to 3G, Supplemental Table 8). Higher baseline LPL (aHR: 0.5 [95% CI: 0.4-0.8], *P* < 0.001) and SCF (aHR: 0.5 [95% CI: 0.4-0.8], *P* = 0.005) values were protective against adverse outcomes while higher baseline MMP12 (aHR: 1.5 [95% CI: 1.1-2.1], *P* = 0.013), CDHR2 (aHR: 1.5 [95% CI: 1.2-2.0], *P* = 0.002), CTSD (aHR: 1.9 [95% CI: 1.2-2.9], *P* = 0.007), GDF-15 (aHR: 1.7 [95% CI: 1.1-2.5], *P* = 0.012), and ACE2 (aHR: 1.8 [95% CI: 1.3-2.6], *P* < 0.001) levels were associated with higher risk of adverse outcomes. Each protein's direction of association was concordant between the HRCP and MACE analyses.

### GSEA pathway analyses for the high-risk composite phenotype

Gene Set Enrichment Analysis (GSEA) was performed using all proteins ranked by *P* values from univariable associations, with HRCP. pathways meeting nominal significance including the KEGG pathways “Renin-angiotensin system” (*P* = 0.001), “Neuroactive ligand-receptor interaction” (*P* = 0.002), “Cytokine-cytokine receptor interaction” (*P* = 0.024), “GnRH signaling pathway” (*P* = 0.039), “Prolactin signaling pathway” (*P* = 0.040), and “Parathyroid hormone synthesis, secretion, and action” (*P* = 0.045) (Supplemental Tables 9 and 10).

### Mendelian randomization for HRCP-associated proteins

Of 37 proteins associated with HRCP, 29 had pQTLs available from published studies and could be assessed using Mendelian randomization.^30^ MLN (*P* < 0.001), MMP12 (*P* = 0.006), and CTSD (*P* = 0.010) were significant in Mendelian randomization testing using inverse-variance weighting, suggesting they are in the causal pathway for CAD. However, only CTSD remained significant after robust Mendelian randomization–Egger analyses, which address horizontal pleiotropy.

### Phenoclustering of clinical variables

We next sought to identify subgroups of similar participants in the PROMISE cohort using *K*-means clustering of clinical variables. The resulting 3 clusters had clear clinical differences: cluster 3 (n = 561) had the lowest prevalence of HRCP (43.0% vs 63.5% in cluster 1 [n = 329), 65.5% in cluster 2 (n = 834]). Cluster 2 had the highest incidence of HRP (13.5% vs 9.0% in cluster 1, 8.2% in cluster 3; Supplemental Table 4). Cluster 3 was the most metabolically healthy, (lowest proportion of hypertension, diabetes, dyslipidemia, and metabolic syndrome) and was largely female. In contrast, cluster 2 was the most metabolically unhealthy cluster (largest proportion of participants with hypertension and diabetes, highest average body mass index, largest percentage of statin users) and was nearly two-thirds male. Cluster 1 had the highest percentage of participants with dyslipidemia and metabolic syndrome along with the highest levels of LDL-C levels, insulin, high-sensitivity troponin I, and triglycerides, and was the youngest on average.

Of the 37 proteins associated with HRCP, 32 proteins differed significantly across phenoclusters. Notably, in cluster 3 (the most metabolically healthy cluster), individuals had the highest levels of LPL and SCF, 2 proteins negatively associated with MACE, as described previously. Four of the 5 proteins positively associated with MACE had their lowest levels of expression in cluster 3 (CDHR2, CTSD, GDF-15, and ACE2), while MMP12 levels did not differ significantly between clusters (Supplemental Table 11).

### Protein score for HRCP in the PROMISE cohort

An elastic net model developed using the 37 HRCP-associated proteins resulted in a protein score that achieved a test set AUC of 0.76 (95% CI: 0.71-0.80). All proteins had nonzero coefficients in the final elastic net model. The protein score was associated with HRCP (aOR: 2.9 [95% CI: 2.2-3.7] per 1 SD increase in score; *P* < 0.001) and with MACE (aHR: 2.7 [95% CI: 1.7-4.2] per 1 SD increase in score; *P* < 0.001; Figure 3H).

Adding the protein score to a clinical model resulted in a statistically significant but only marginally improved discrimination by an AUC of 0.02 for discrimination of HRCP (AUC: 0.77 [95% CI: 0.74-0.79] for clinical model to 0.79 [95% CI: 0.77-0.82] for clinical + protein score; *P* < 0.001; Supplemental Figure 6). To further assess incremental value beyond AUC, net reclassification index (NRI) analysis was performed using tertiles of predicted risk of presence of HRCP. The addition of the protein score to the clinical model yielded a categorical NRI of 0.13 (95% CI: 0.08-0.18; *P* < 0.001) and an integrated discrimination index (IDI) of 0.04 (95% CI: 0.03-0.05; *P* < 0.001), indicating significant, moderate improvement in reclassification for HRCP when adding the protein score to a clinical model (Supplemental Table 12).

### Dan-NICAD validation cohort

**Figure 4:**
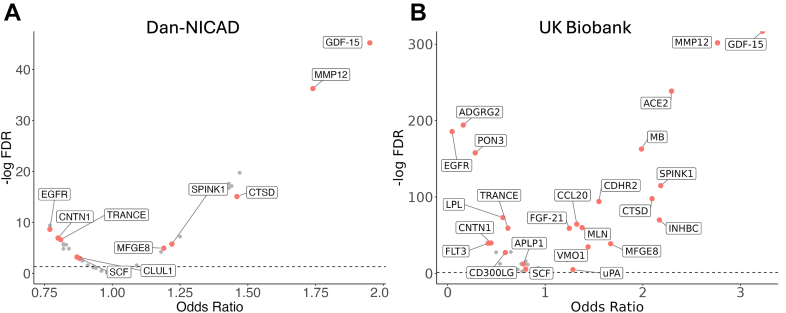
Association of Proteins With HRCP in Dan-NICAD and With Prevalent CAD in UK Biobank Volcano plots of protein associations with HRCP in Dan-NICAD (A) and with CAD in UK Biobank (B). Plots show odds ratios and FDR-adjusted *P* values from univariable logistic regression. The dotted line represents FDR cutoff significance in univariable logistic regression models (*q* < 0.05). Highlighted red dots correspond to the proteins that remain significant in multivariable analyses (*P* < 0.05). CAD = coronary artery disease; FDR = false discovery rate; HRCP = high-risk composite phenotype.

Of the 37 proteins associated with HRCP in PROMISE, 30 proteins (81%) were validated for association with HRCP in univariable analyses in Dan-NICAD (*q* < 0.05). Ten proteins, including CTSD, GDF-15, MMP12, and SCF, remained significant in multivariable analyses with direction of effect consistent with PROMISE (Figure 4A, Supplemental Table 13). The PROMISE elastic net-based protein score was significantly associated with HRCP (aOR: 1.3 [95% CI: 1.2-1.4], *P* < 0.001) and demonstrated an AUC of 0.71 (95% CI: 0.69-0.73) for HRCP in Dan-NICAD. Similar to the findings in PROMISE, the addition of the protein score only marginally improved the predictive performance of a clinical-only model from an AUC of 0.80 (95% CI: 0.79-0.82) to 0.81 (95% CI: 0.79-0.82; *P* = 0.011) (Supplemental Figure 7).

### UK Biobank validation cohort

All 37 HRCP proteins were associated with prevalent CAD in UKB (N = 53,018) in univariable analyses (*q* < 0.05), and 24 of the proteins remained associated with CAD in multivariable analyses (*P* < 0.05) (Figure 4B, Supplemental Table 14). Nine of 10 proteins significant in multivariable analyses from the Dan-NICAD dataset were also validated in the UKB dataset (CNTN1, CTSD, EGFR, GDF-15, MFGE8, MMP12, SCF, SPINK1, and TNFSF11).

The elastic net-based PROMISE protein score was also significantly associated with prevalent CAD (aOR: 1.9 [95% CI: 1.8-2.1], *P* < 0.001) in UKB, yielding an AUC of 0.78 (95% CI: 0.78-0.79) in UKB. A clinical model including the same features used in PROMISE was trained on the UKB dataset and had an AUC of 0.865 (95% CI: 0.86-0.87), which only marginally improved (AUC: 0.873, 95% CI: 0.87-0.88, *P* < 0.001) when the protein score was added (Supplemental Figure 8).

Among 50,450 participants without prevalent CAD, 4,342 participants (8.6%) developed CAD over a mean follow-up of 13.2 (SD: 2.3) years. The majority of proteins (36 of 37) were associated with time-to-CAD, and 26 of these remained significant in multivariable analyses, including CTSD (Supplemental Tables 15 and 16). Results adjusting for proportional hazards violations (see Methods) were generally consistent with the overall model (Supplemental Tables 15 and 16). The elastic net protein score was also significantly associated with incident CAD (aHR: 1.61 [95% CI: 1.52-1.69]; *P* < 0.001) in multivariable analysis.

### Protein associations for high-risk plaque in PROMISE

We conducted exploratory analyses in PROMISE to identify proteins uniquely reporting on HRP alone. Four proteins were associated with HRP in univariable analyses (*q* < 0.05), including LEP, CGB3, PSPN, and VAT1. Only VAT1 remained significant in multivariable analyses (*P* < 0.05) (Supplemental Figure 9, Supplemental Table 17). In sensitivity analyses excluding individuals with oCAD, 29 proteins were nominally (*P* < 0.05) associated with HRP, but none survived adjustment for clinical covariates or FDR correction (*q* < 0.05) (Supplemental Figure 10).

## Discussion

By integrating CCTA phenotyping with proteomic profiling across 3 large cohorts, we identified circulating biomarkers linked to atherosclerosis imaging characteristics not routinely captured. We found that individual proteins and a 37-protein risk score were associated with HRCP and MACE. Further, these proteins, which corresponded with distinct patient phenoclusters, report on well-established pathways such as inflammation and metabolic regulation and less-characterized protein pathways including those involved in protein degradation, cell adhesion, and extracellular matrix remodeling, and neuroactive ligand-receptor interactions. While the protein score added little discrimination beyond clinical risk factors, it moderately improved reclassification, suggesting that proteomic profiles may complement clinical models. Mendelian randomization provided causal evidence for cathepsin D (CTSD) as a candidate therapeutic target for CAD, underscoring the biological value of these findings.

In addition to the protein score, 9 individual proteins were consistently associated with HRCP or CAD across 3 studies: the higher risk symptomatic PROMISE and Dan-NICAD cohorts, as well as the healthier UKB population-based cohort. These proteins include CNTN1, CTSD, EGFR, GDF-15, MFGE8, MMP12, SCF, SPINK1, and TNFSF11, most of which have known associations with atherosclerosis. CNTN1 (contactin 1) and MMP12 (matrix metallopeptidase) have emerged as potential markers for atherosclerosis, although the precise molecular mechanisms remain to be elucidated.^38^^,^^39^ EGFR (epidermal growth factor receptor) and MFGE8 (milk fat globule-EGF factor 8 protein) are both increased in atherosclerotic tissue and have been associated with increased smooth muscle cell proliferation.^40^^,^^41^ GDF-15, a stress-responsive cytokine highly expressed by macrophages in plaques, has also been previously associated with atherosclerotic cardiovascular disease and cardiovascular death for heart failure.^42^ SCF (stem cell factor) is a cytokine shown to improve survival after myocardial infarction in mice, likely due to attenuating cardiac remodeling.^43^ To our knowledge, SPINK1 (serine protease inhibitor Kazal-type 1) has not been associated with atherosclerosis. TNFSF11 (tumor necrosis factor superfamily member 11) has been shown to increase vascular calcification via activation of the NF-κB pathway.^44^

In PROMISE, 7 of the 37 HRCP-associated proteins were also significantly associated with MACE. Higher levels of LPL and SCF were protective, associated with lower odds of HRCP, lower risk of adverse cardiac events, and cluster 3 (participants who had the lowest percentage of HRCP or HRP), consistent with their known roles in lipolysis and cardiac remodeling.^45^ In contrast, higher levels of ACE2, MMP12, GDF-15, CDHR2, and CTSD were associated with increased risk of MACE and HRCP. While most of these have plausible biological links to cardiovascular disease, to our knowledge, no association between CDHR2 and cardiovascular disease has been reported in the literature.^46^

CTSD (cathepsin D) emerged as a strong candidate from our study, as higher levels were associated with HRCP across all 3 studies, with higher risk of incident events in PROMISE and UKB and higher levels in the metabolically unhealthy phenoclusters. Further, Mendelian randomization analyses demonstrated that CTSD is in the causal pathway of CAD, rather than being a byproduct of the disease. High plasma levels of CTSD have previously been tied to cardiovascular events.^47^ Cathepsin D is a protease ubiquitous within lysosomes and plays a role in degradation of apolipoprotein B.^48^ Its modification of LDL particles promotes foam cell formation and contributes to lipid accumulation in the arterial intima with increased LDL buildup. The oxidized LDL enhances inflammation, creating a feedback loop triggering release of more cathepsin D, which also adversely promotes smooth muscle migration and proliferation, leading to adverse remodeling and progression of atherosclerosis.^49^ Consistent with these findings, extracellular matrix proteomics of arterial tissue has identified cathepsin D as part of a molecular signature associated with symptomatic carotid plaques and long-term cardiovascular outcomes.^50^ Although no cathepsin D inhibitors are currently in clinical trials, CTSD is a potentially druggable gene that can be targeted by approved small molecules and biotherapeutics.^51^

Notably, while the individual proteins and the protein score were all independently associated with HRCP in multivariable models, the protein score statistically significantly but only clinically minimally improved discrimination of HRCP over a clinical model (increase in AUC of 0.02), in part because of the high discrimination of a relatively simple clinical model. NRI analyses, which are more sensitive to reclassification than AUC alone, demonstrated not only a statistically but also a more clinically meaningful significant reclassification when the protein score was added to a clinical model (categorical NRI = 0.13, *P* < 0.001; IDI = 0.04, *P* < 0.001). These improvements are within the range reported for other cardiovascular biomarkers; for example, the addition of high-sensitivity troponin I to the pooled cohort equations yielded categorical NRIs of 0.03 to 0.09 for atherosclerotic cardiovascular disease, global cardiovascular disease, and heart failure, and the combination of high-sensitivity troponins I and T yielded a categorical NRI of 0.12 for heart failure.^52^ Similarly, the addition of a CAD polygenic risk score to the pooled cohort equations yielded a categorical NRI of 0.04 for incident CAD.^53^ While these results suggest clinical utility of the identified proteins in addition to their biologic significance, it is important to note that direct comparison of NRI values across studies is limited by differences in risk thresholds, baseline model calibration, and outcome definitions.^54^

Our study has several important limitations. One of our objectives was to identify circulating biomarkers associated with high-risk plaque. Although we identified only 1 protein, VAT1 (vesicle amine transport 1) as associated with HRP, it did not remain significant after excluding patients with oCAD. While our study is one of the largest of its kind, these analyses were limited by our small population set, with only 160 participants having at least 1 HRP feature. Second, CCTA-based imaging phenotypes are not available in UKB; although CAD overlaps significantly with HRCP, it is a separate, broader phenotype. Third, our discovery and validation cohorts were predominantly composed of individuals self-reporting as White race, which may limit the generalizability to populations with greater racial and ethnic diversity. Additionally, we used the Olink platform, which provided a broad assay of hundreds of proteins. However, no proteomics platform captures all circulating proteins relevant to atherosclerosis. Notably, this platform does not capture individual apolipoproteins including the clinically important ApoB. While our models adjust for LDL-C, which is highly correlated with ApoB, the absence of apolipoproteins from the protein score and the clinical model should be noted as a limitation. Residual confounding by age or unmeasured medications beyond statins cannot be fully excluded, although consistent replication across 3 cohorts with different age distributions and enrollment criteria mitigates this concern. Lastly, proteomic profiling was performed at a single baseline time point, and thus we do not capture the dynamic changes in protein levels over time.

Overall, although our results did not identify proteins with clinical utility for discriminating patients with HRCP, they have robustly highlighted several novel pathways and markers underlying CAD and HRCP. The biomarkers identified help define molecular phenotypes, were associated with future cardiovascular events, and delineated distinct patient phenoclusters with different risk profiles. Furthermore, our identification of CTSD as a protein with a causal role in CAD pathophysiology underscores its promise as a novel, druggable therapeutic target.

### Data Availability

PROMISE clinical data sets are available at https://biolincc.nhlbi.nih.gov/studies/promise. There are no commercial use data restrictions, and no data restrictions based on area of research. The molecular data will be stripped of identifiers and deposited in dbGAP per NIH policy (up to 6 months after data submission is initiated or at the time of acceptance of initial publication using these data, whichever occurs first).

## Funding Support and Author Disclosures

The project described was supported by the National Heart, Lung, and Blood Institute (grant No. 1R01HL098237, 1R01HL098236, 1R01HL146145-01A1, 1R01HL098305). The content is solely the responsibility of the authors and does not necessarily represent the official views of the National Institutes of Health. Dr Kwee reports funding through sponsored research agreements with Duke from Baseline Study LLC, AstraZeneca, and Lilly. Dr Ferencik reports consulting fees from Cleerly, Elucid, Heart Flow, and Biomarin; serves on the advisory board of Cleerly and Elucid; and has stock options in Elucid. Dr Helgason is an employee of deCODE Genetics/Amgen, Reykjavik, Iceland. Dr Foldyna reports unrelated research grant support from AstraZeneca, MedImmune, MedTrace, Ionis, and Cleerly. Dr Shah reports funding through sponsored research agreements with Duke from Baseline Study LLC, AstraZeneca, and Lilly; Dr Shah also holds 2 unlicensed patents on which she is listed as a coinventor on a metabolomics finding. All other authors have reported that they have no relationships relevant to the contents of this paper to disclose.

Perspectives **COMPETENCY IN MEDICAL KNOWLEDGE:** Proteomic profiling in symptomatic patients undergoing coronary computed tomography angiography identifies circulating signatures linked to high-risk plaque and incident events but adds little discrimination beyond simple clinical risk models. Clinicians should recognize that broad multiprotein panels may not meaningfully improve near-term risk stratification in this setting, and that their current value lies primarily in clarifying underlying biology, including inflammatory, metabolic, and proteolytic pathways such as those involving cathepsin D.

**TRANSLATIONAL OUTLOOK:** These findings support a translational focus on using plasma proteomics to map atherosclerotic plaque biology and prioritize therapeutic targets rather than to build complex prediction tools for symptomatic patients already selected for imaging. Future work should include experimental studies that directly test whether modulation of cathepsin D and related pathways alters plaque composition and event risk, as well as prospective evaluations of more targeted proteomic panels, alternative populations, and serial sampling strategies that may yield greater clinical utility.
