# Supplementary material for: Proteomic Signatures of High-Risk Coronary Plaque Features and Incident Events
Source: JACC Basic Transl Sci. 2026 Jul 22;11(8):101640. doi: 10.1016/j.jacbts.2026.101640 (PMC13416469; doi:10.1016/j.jacbts.2026.101640)
Supplement: Supplemental Methods, Supplemental Figures 1 to 10, and Supplemental Tables 1 to 17 [file mmc1.docx]

**Supplementary Material**

**Table of Contents**

**Supplemental Methods**

- PROMISE Laboratory Methods
- UK Biobank Outcomes
- Statistical Analysis

**Supplemental Figures**

- **Supplemental Figure 1:** Cohort diagram of datasets used in manuscript: PROMISE, Dan-NICAD, and UK Biobank.
- **Supplemental Figure 2:** Association of proteins with Leaman score in PROMISE.
- **Supplemental Figure 3:** Association of proteins with high CAC in PROMISE.
- **Supplemental Figure 4:** Association of protein levels with oCAD in PROMISE.
- **Supplemental Figure 5:** Association of protein levels with HRP without oCAD in PROMISE.
- **Supplemental Figure 6:** Receiver Operating Characteristic (ROC) curves for models trained on PROMISE dataset.
- **Supplemental Figure 7:** Receiver Operating Characteristic curves in the Dan-NICAD dataset.
- **Supplemental Figure 8:** Receiver Operating Characteristic (ROC) in the UK Biobank with prevalent CAD.
- **Supplemental Figure 9:** Association of protein levels with HRP in PROMISE.
- **Supplemental Figure 10:** Association of proteins with HRP without oCAD in PROMISE.

**Supplemental Tables**

- **Supplemental Table 1:** Baseline Characteristics of the Dan-NICAD Cohort
- **Supplemental Table 2:** Baseline Characteristics of the UK Biobank Cohort
- **Supplemental Table 3:** UK Biobank ICD codes for CAD
- **Supplemental Table 4:** Summary Statistics for Clinical Variables across Phenoclusters
- **Supplemental Table 5:** Clinical characteristics of PROMISE proteomics substudy and overall parent clinical trial
- **Supplemental Table 6:** Results of Univariable Models for Association of Proteins with HRCP in the PROMISE cohort
- **Supplemental Table 7:** Results of Multivariable Models for Association of Proteins with HRCP in the PROMISE cohort
- **Supplemental Table 8:** Cox Time-to-MACE Models in the PROMISE Cohort
- **Supplemental Table 9:** Gene Set Enrichment Analyses for HRCP (Based on Univariable HRCP Models, Top 10 Hallmark Pathways Displayed)
- **Supplemental Table 10:** Gene Set Enrichment Analyses for HRCP (Based on Univariable HRCP Models, Top 10 KEGG Pathways Displayed)
- **Supplemental Table 11:** Protein Levels Differences Between Phenoclusters
- **Supplemental Table 12:** Dan-NICAD validation protein association with HRCP
- **Supplemental Table 13:** UK Biobank validation protein association with prevalent CAD
- **Supplemental Table 14:** UK Biobank Time-to-Incident CAD Event Cox Models
- **Supplemental Table 15:** Association between protein levels and time to incident CAD in UK Biobank time-stratified models
- **Supplemental Table 16:** PROMISE validation protein association with HRP

**Supplemental Methods**

***PROMISE Laboratory Methods***

Olink based proteomic profiling was performed in PROMISE and was available in the Dan-NICAD and UK Biobank validation cohorts. In the PROMISE study, biospecimens were collected via peripheral phlebotomy in a subset of clinical sites (N=4,017, 40.2% of overall trial participants) and immediately processed for plasma and samples frozen and stored at -80^o^C. For this study, N=1,805 individuals with both biospecimens and CCTA images were used. Proteomic profiling was conducted on thawed plasma using Olink Target 96 platforms (Olink Biosciences, Uppsala, Sweden), which employs Proximity Extension Assay (PEA) technology for high-throughput multiplex immunoassays, yielding log2-transformed normalized protein expression (NPX) values for relative protein quantification. Each Olink Target panel contains 92 proteins; for this study, seven Olink panels were profiled (CVD II and III, Development, Inflammation, Metabolism, Oncology III, and Cell Regulation panels, N=644 proteins). After preprocessing and normalization, QC of the data included principal components analysis to identify outliers, examination of control assay results, and diagnostic visualizations. In addition, we excluded any sample (N=81 samples) that lost an entire panel at the laboratory QC step – either due to complete assay failure or blank runs manually flagged by analysts. Assays were categorized based on the percentage of samples below the limit of detection (LOD): those with ≤25% below LOD were treated as continuous variables; assays with 25-75% below LOD were dichotomized as detected vs. undetected; and assays with ≥75% below LOD (N=55) were excluded from further analysis. Fifteen proteins were assayed on more than one panel; for each of these, we retained data from a single panel, choosing the version with the lowest number of observations below LOD. Finally, we removed two proteins for which diagnostic plots suggested the proteins were too concentrated to be accurately assayed. For sporadic missing data points, median imputation was applied. Overall, the final dataset after QC included 1,724 samples and 572 unique proteins (502 analyzed as continuous, 70 assays dichotomized as detected vs. undetected).

***UK Biobank Outcomes***

In UK Biobank, since CCTA imaging is not available, prevalent CAD was used as the primary outcome, defined as CAD that was diagnosed prior to enrollment, derived from self-report, electronic health record (EHR) data, and death registry data. For self-report, participants were considered to have CAD if they reported a medical history of “heart attack/myocardial infarction” or “heart attack” or reported any of the following operations: “coronary angioplasty (PTCA) +/- stent”, “coronary artery bypass grafts (CABG)”, or “triple heart bypass.” For the EHR and death registry data, eligible ICD9/ICD10 codes are given in **Supplemental Table 3**. CAD controls were defined as no CAD at time of enrollment. Incident CAD was defined as having one of the above CAD diagnoses after the enrollment visit, or having a CAD ICD code recorded in the death registry and no prevalent CAD. For time-to-event analyses, time to incident CAD was defined as the time between the enrollment visit and the first eligible CAD code, with data censored on October 31, 2022.

***Statistical Analysis***

The overall analytic approach involved discovery analyses in PROMISE, followed by validation in Dan-NICAD and UK Biobank. In PROMISE, we first identified proteins associated with HRCP; tested proteins for association with MACE; performed pathway analysis, Mendelian randomization to infer causality with CAD, phenoclustering of participants based on clinical and imaging data; and developed a protein-based score for HRCP discrimination using elastic net penalized regression. Individual protein associations and the protein score were validated in Dan-NICAD (HRCP) and UKB (prevalent and incident CAD). Secondary outcomes examined included protein associations with individual HRCP components and HRP without oCAD. False Discovery Rate (FDR) adjustment (q<0.05) was applied for initial protein discovery steps with subsequent individual protein explorations using a nominal p<0.05 as specified below.

In the PROMISE discovery cohort, each of 572 proteins was first tested for association with HRCP using univariable logistic regression models, adjusted for multiple comparisons. To quantify the clinical risk captured by HRCP, we tested HRCP status for association with time-to-incident MACE using Cox proportional hazards models with and without adjustment for baseline clinical covariates (age, sex, self-reported race, BMI, LDL-C, diabetes, hypertension, statin use, and smoking status). Kaplan–Meier curves were used to estimate cumulative MACE incidence by HRCP status at prespecified time horizons (1, 2, 3, and 5 years). Proportional hazards assumptions were checked and were not violated for HRCP. Proteins significant in univariable analyses were then tested in multivariable analyses (adjusted for age, sex, self-reported race, body mass index (BMI), low-density lipoprotein-cholesterol (LDL-C), diabetes, hypertension, statin use, and smoking status, and these significant proteins were subsequently tested for association with time-to-MACE in PROMISE using Cox proportional hazard models (adjusted for same covariates as HRCP). Proportional hazards assumptions were checked, and analyses were stratified by age (> median vs. ≤ median) and LDL (>160 vs. ≤160 mg/dL) to address violations. Pathway analysis was conducted on all proteins associated with HRCP from univariable analyses (q < 0.05) using Gene Set Enrichment Analysis (GSEA), performed using 195 KEGG and 46 Hallmark gene sets ([Liberzon et al., 2015](#_ENREF_22); [Liberzon et al., 2011](#_ENREF_23); [Subramanian et al., 2005](#_ENREF_35)). GSEA utilized the fgsea package in R with a scoreType of "pos" and a minimum pathway size of 3 ([Korotkevich et al., 2021](#_ENREF_20)). As a sensitivity analysis, FDR correction was also applied to the multivariable results across all 572 proteins.

Two sample cis-Mendelian randomization was conducted using protein quantitative trait loci (pQTLs) identified using a previously published genome-wide association study (GWAS) meta-analyses of CAD due to a lack of genetic studies specifically assessing HRCP ([van der Harst & Verweij, 2018](#_ENREF_36)). Genetic instruments for each protein were chosen using a relaxed threshold of <1x10^-6^ (because of lack of significant pQTLs with a more conservative significance) and linkage disequilibrium r^2^ = 0.4. Causal estimates were derived using the inverse-variance weighted (IVW) method for multiple pQTLs and the Wald ratio method for single pQTLs. Prior to Mendelian randomization, we conducted heterogeneity testing with Steiger filtering and outlier analyses to check for pleiotropy and multicollinearity. MR Egger regression was used to assess for horizontal pleiotropy (i.e. when genetic variants influence multiple traits in different biological pathways).

Phenoclustering was performed using K-means clustering to discern latent structures, i.e. groups of patients with similar features. Input variables consisted of a large set of clinical and imaging variables as listed in **Supplemental Table 4.** Imputation of missing data was performed using median imputation for continuous variables and mode imputation for discrete variables. Based on within-cluster sum of squares (WSS) in a scree plot, we determined the optimal number of clusters to be three. ANOVA models were then used to test for differences in clinical variables between the identified clusters, followed by pairwise Tukey’s tests for comparison of clusters for significant variables (p < 0.05).

To develop a predictive protein score for HRCP, an elastic net penalized regression model was trained in the PROMISE cohort. The input variables for this model were the 37 proteins associated with HRCP in PROMISE multivariable models. The data were split into 70% training and 30% test sets, with a balanced presence of HRCP in both sets. The glmnet package was used through the caret framework in R to implement elastic net training. Model tuning involved fivefold cross-validation with grid search for alpha (0 to 1, 0.1) and lambda (10^-4^ to 10^0^, 100 values). The coefficients from the optimal model (selected based on cross-validation performance) define the HRCP protein score. The performance of this protein-only model was assessed using the area under the receiver operating characteristic (ROC) curve (AUC) on the held-out test set. The association between the calculated protein score and HRCP (in the test set) was tested using logistic regression, and its association with MACE was tested using multivariable Cox models (adjusting for clinical covariates). For comparison, standard, unpenalized logistic regression models were fit using: (1) only clinical covariates (sex, minority race, age, LDL-C, BMI, statin use, smoking status, hypertension, diabetes) and (2) the clinical covariates plus the elastic net-derived protein score as a single additional predictor variable. Model performance was compared using AUCs and the DeLong Test. To further assess incremental value of the protein model over a clinical model for HRCP, net reclassification improvement (NRI) and integrated discrimination improvement (IDI) were calculated comparing the clinical-only model to the clinical plus protein score model, using tertiles of predicted HRCP risk from the clinical model as cutpoints for categorical NRI.

Exploratory analyses similarly tested protein association (univariable and multivariable logistic regression) with individual HRCP components (oCAD, CAC >400, HRP features, Leaman >5) and with HRP without oCAD in the PROMISE cohort.

Validation analyses were performed in the Dan-NICAD cohort using the 37 proteins associated with HRCP in PROMISE multivariable models using univariable and multivariable logistic regression models (adjusting for similar covariates). The HRCP protein score derived from PROMISE was calculated for Dan-NICAD participants and tested for association with HRCP using logistic regression. Its discriminative ability for HRCP was assessed using AUC. Performance of a clinical-only logistic regression model for HRCP was compared to a model including clinical covariates plus the PROMISE-derived protein score using AUC. In UKB, these 37 proteins and the protein score were tested for association with prevalent CAD using univariable and multivariable logistic regressions (adjusting for similar covariates). The PROMISE-derived HRCP protein score was tested for association with prevalent CAD using logistic regression and its AUC for discriminating prevalent CAD was calculated. Performance of a clinical-only logistic regression model for prevalent CAD was compared to a model including clinical covariates plus the PROMISE-derived protein score using AUC. Associations between the 37 PROMISE HRCP proteins and the protein score and time-to-incident CAD were assessed using multivariable Cox proportional hazard models. For proteins that showed evidence of proportional hazards violations, stratified Cox models were created by time from enrollment (0-5, 5-10, and >10 years), tested again for proportional hazards violations, and report interval-specific hazard ratios and confidence intervals where appropriate.

**Supplemental Figure 1:** Cohort diagram of datasets used in manuscript: PROMISE, Dan-NICAD, and UK Biobank.


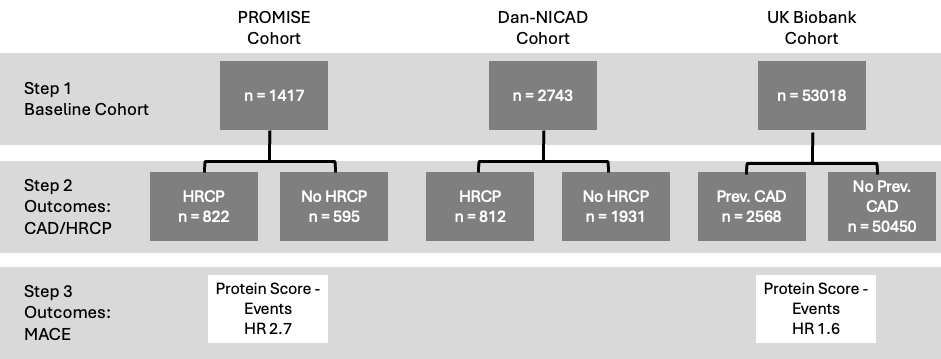


**Supplemental Figure 2: Association of proteins with Leaman score in PROMISE.** Forest plot showing the odds ratio (OR) and 95% confidence intervals in the PROMISE trial for the association between Leaman score >5 and the 37 proteins previously found to be associated with HRCP. Multivariable odds ratio (and 95% confidence interval) plotted for proteins significant (p < 0.05) in univariable analyses in logistic regression models.

**
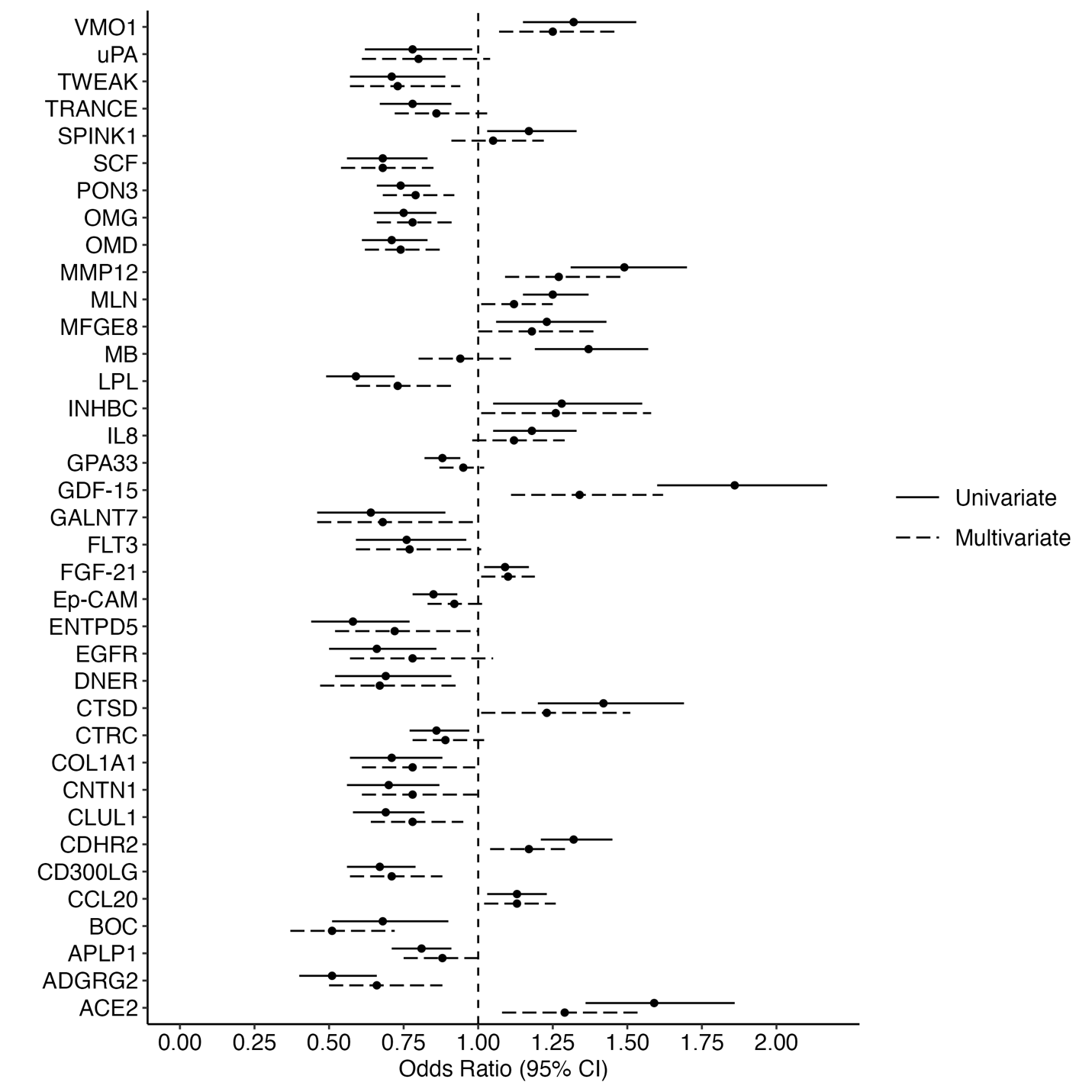
**

**Supplemental Figure 3: Association of proteins with high CAC in PROMISE.** Forest plot showing the odds ratio (OR) and 95% confidence intervals in the PROMISE trial for the association between CAC score >400 and the 37 proteins previously found to be associated with HRCP. Multivariable odds ratio (and 95% confidence interval) plotted for proteins significant (p < 0.05) in univariableunivariable analyses in logistic regression models.

**
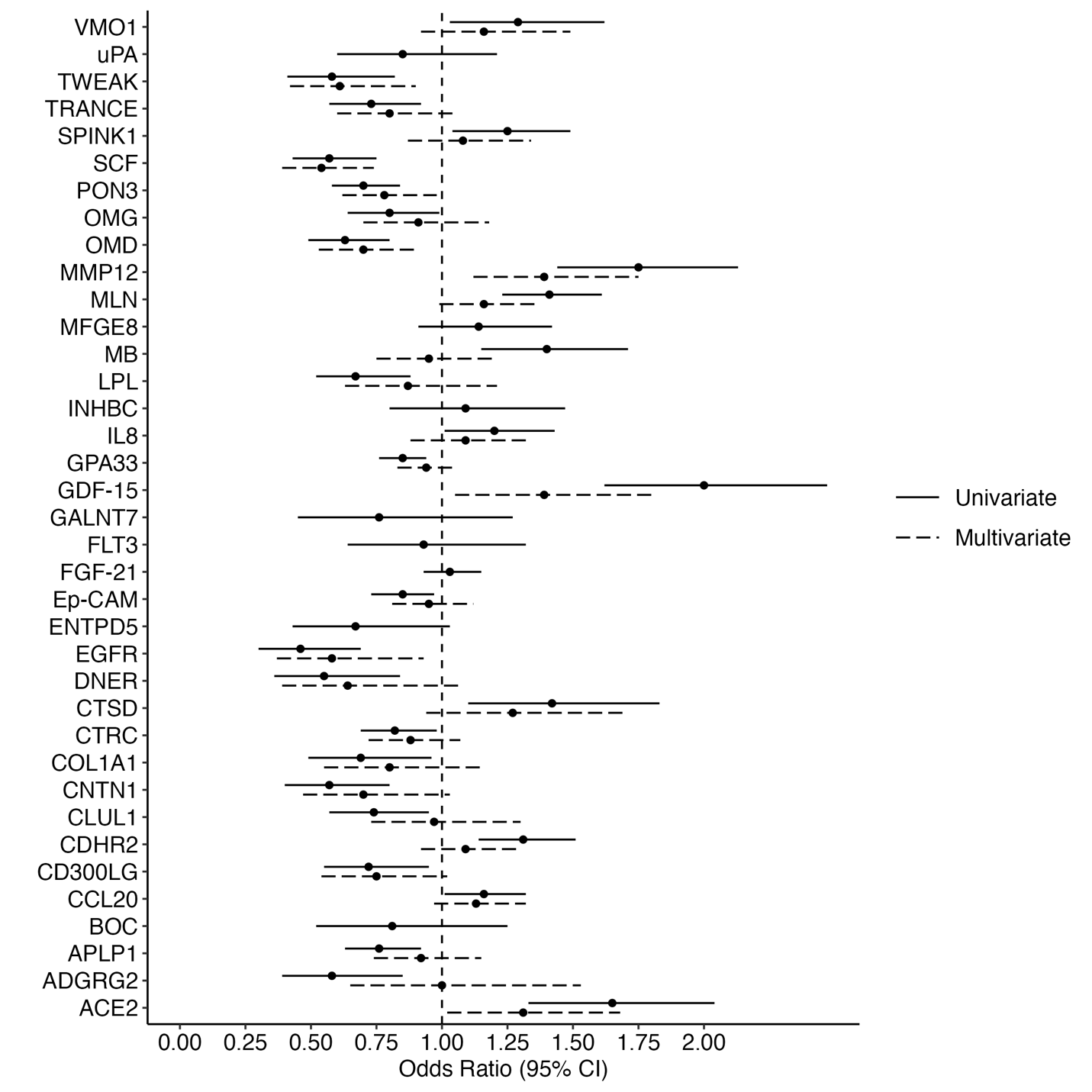
**

**Supplemental Figure 4: Association of protein levels with oCAD in PROMISE.** Forest plot showing the odds ratio (OR) and 95% confidence intervals in the PROMISE trial for the association between oCAD ≥ 50% stenosis and the 37 proteins previously found to be associated with HRCP. Multivariable odds ratio (and 95% confidence interval) plotted for proteins significant (p < 0.05) in univariableunivariable analyses in logistic regression models.

**
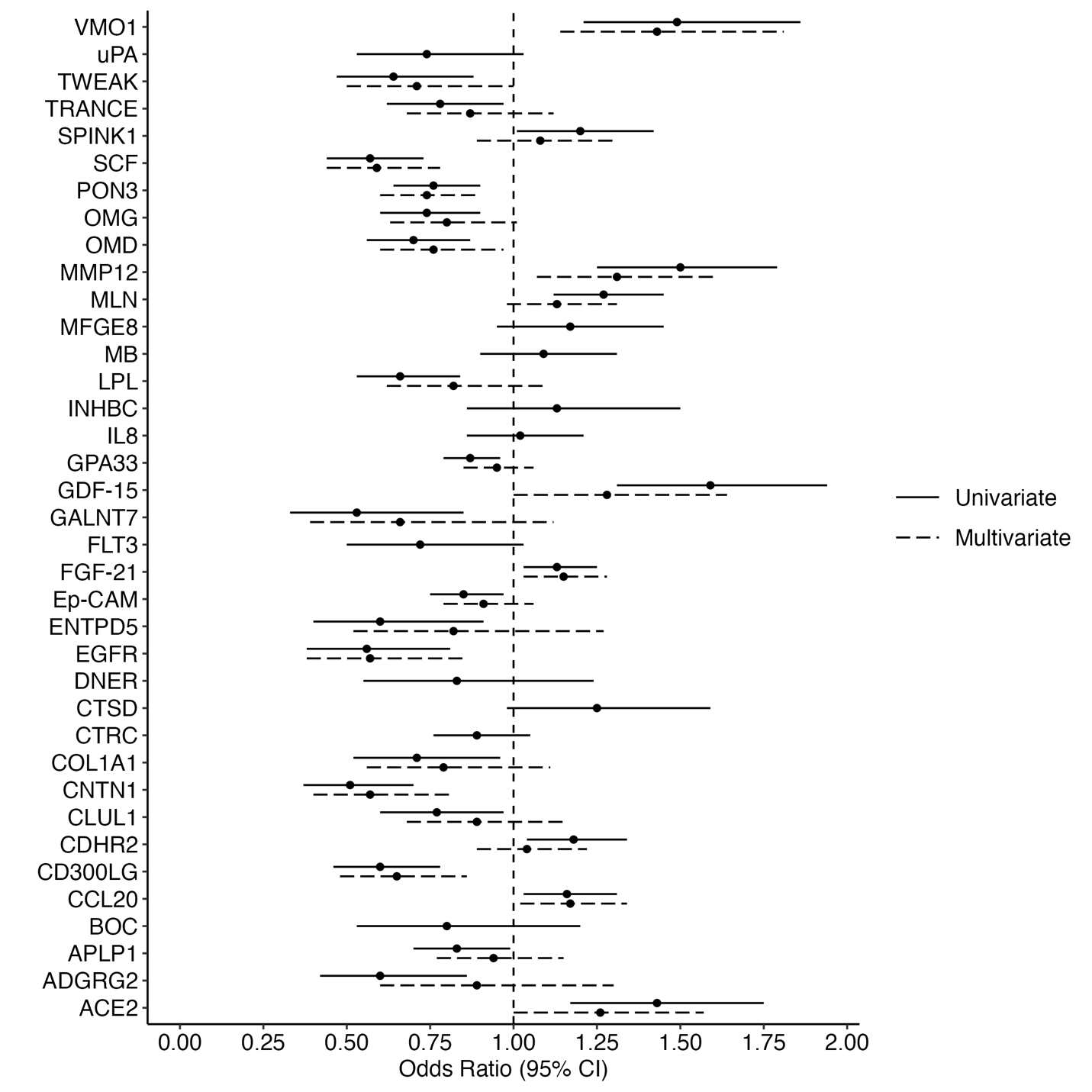
**

**Supplemental Figure 5: Association of protein levels with HRP without oCAD in PROMISE.** Forest plot showing the odds ratio (OR) and 95% confidence intervals in the PROMISE trial for the association between HRP in patients without oCAD and the 37 proteins previously found to be associated with HRCP. Multivariable odds ratio (and 95% confidence interval) plotted for proteins significant (p < 0.05) in univariableunivariable analyses in logistic regression models.


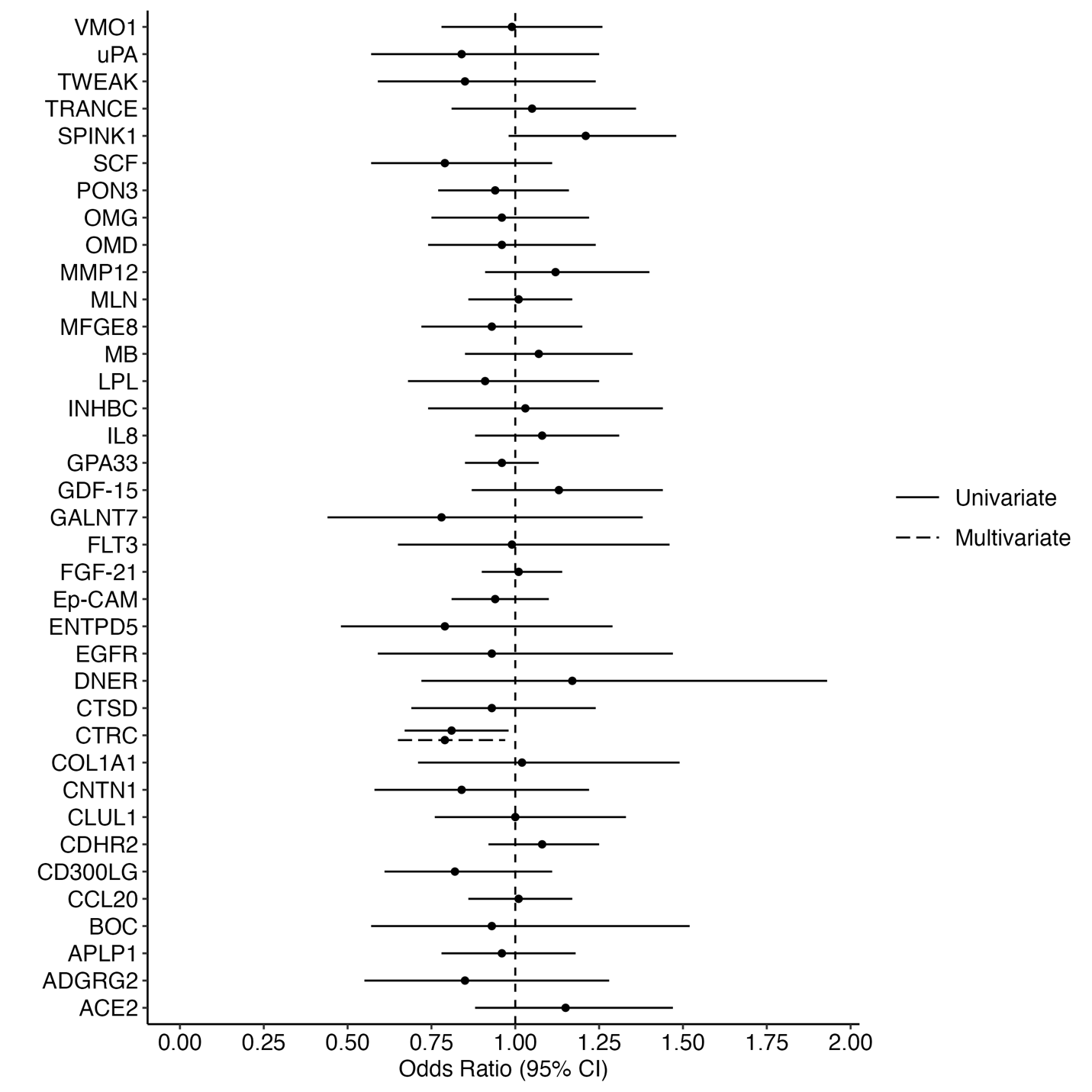


**Supplemental Figure 6: Receiver Operating Characteristic (ROC) curves for models trained on PROMISE dataset.** ROC curves compare performance in predicting HRCP of a clinical-only logistic regression model and a combined model that incorporates both the clinical covariates and an elastic net-based protein score,


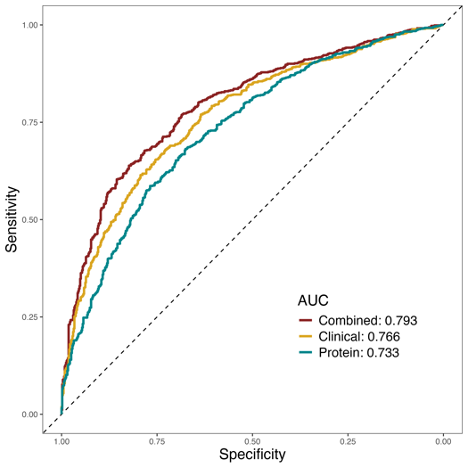


**Supplemental Figure 7: Receiver Operating Characteristic curves in the Dan-NICAD dataset.** ROC curves show performance in predicting HRCP of PROMISE-derived protein score when applied to the Dan-NICAD cohort. The plot compares a clinical-only logistic regression model using Dan-NICAD covariates against a model combining the Dan-NICAD clinical factors with the externally developed protein score.


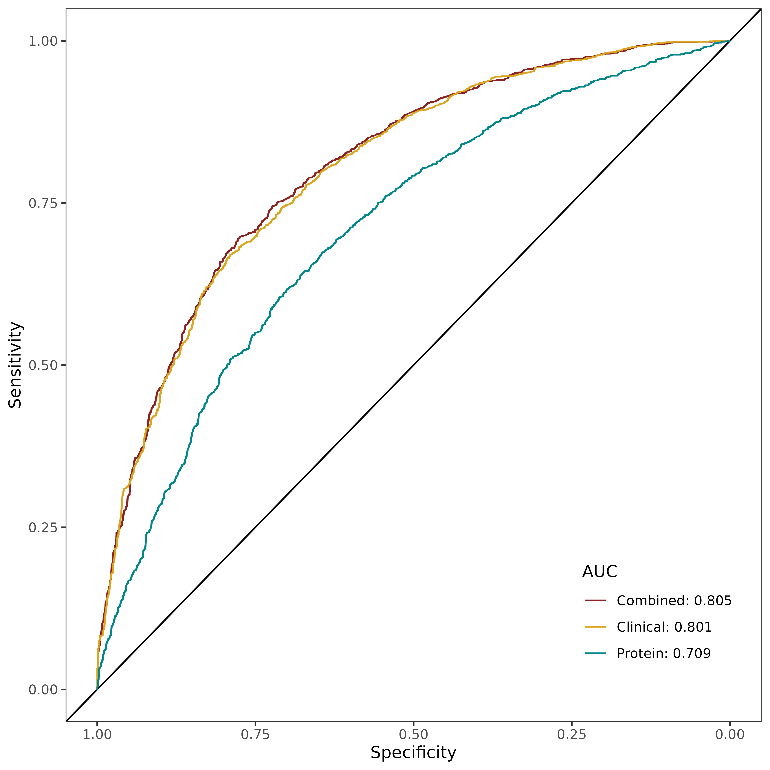


**Supplemental Figure 8: Receiver Operating Characteristic (ROC) in the UK Biobank with prevalent CAD.** ROC curves show performance in predicting CAD of PROMISE-derived protein score when applied to the UK Biobank cohort. The plot compares a clinical-only logistic regression model using UK Biobank covariates against a model combining the UK Biobank clinical factors with the externally developed protein score.


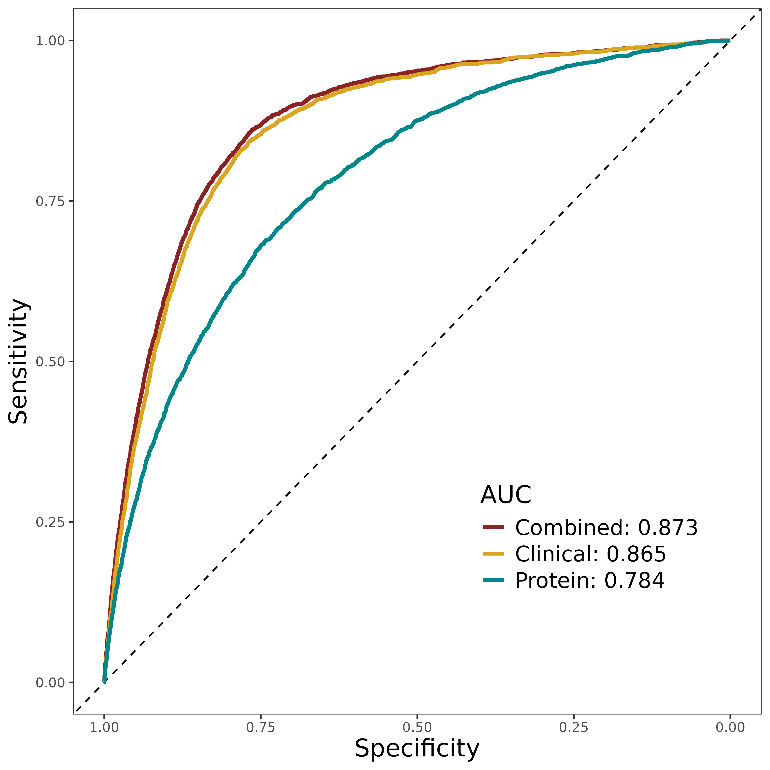


**Supplemental Figure 9: Association of protein levels with HRP in PROMISE.** Volcano plot with odds ratios (X-axis) and FDR adjusted p-values (Y-axis) derived from univariableunivariable logistic regression models for HRP. The dotted line represents FDR cutoff significance in univariableunivariable logistic regression models (q < 0.05). Red dots correspond to the proteins that remain significant in multivariable analyses (p < 0.05).

**
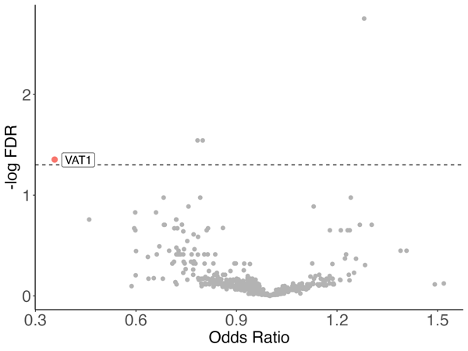
**

**Supplemental Figure 10: Association of proteins with HRP without oCAD in PROMISE.**

Volcano plot with odds ratios (X-axis) and FDR adjusted p-values (Y-axis) derived from univariableunivariable logistic regression models for HRP without oCAD. The dotted line represents FDR cutoff significance in univariableunivariable logistic regression models (q < 0.05). No proteins remained significant in multivariable analyses (p < 0.05).


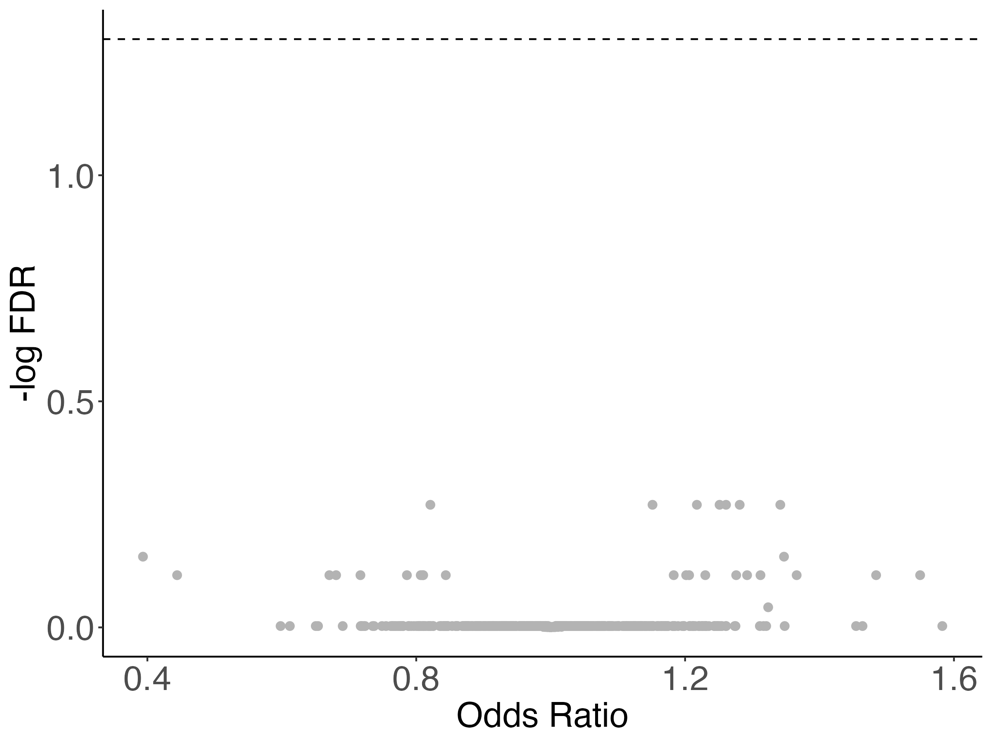


**Supplemental Table 1. Baseline Characteristics of the Dan-NICAD Cohort**

|  | **HRCP**  **(N=1356)** | **No HRCP**  **(N=1387)** | **Overall**  **(N=2743)** |
| --- | --- | --- | --- |
| **Age (years), mean (SD)** | 61.5 (8.1) | 54.5 (9.2) | 58.0 (9.3) |
| **Self-Reported Race, n (%)** |  |  |  |
| White | 1346 (99.3%) | 1371 (98.8%) | 2717 (99.1%) |
| Other | 10 (0.7%) | 16 (1.2%) | 26 (0.9%) |
| **Female sex, n (%)** | 467 (34.4%) | 832 (60.0%) | 1299 (47.4%) |
| **Diabetes, n (%)** | 120 (8.8%) | 51 (3.7%) | 171 (6.2%) |
| **Hypertension, n (%)** | 646 (47.6%) | 411 (29.6%) | 1057 (38.5%) |
| **LDL-C (mmol/L), mean (SD)** | 3.10 (1.004) | 3.16 (0.908) | 3.13 (0.957) |
| **BMI (kg/m^2^), mean (SD)** | 27.5 (4.13) | 27.0 (4.41) | 27.2 (4.28) |
| **Smoker, n (%)** | 858 (63.3%) | 637 (45.9%) | 1495 (54.5%) |
| **Cholesterol Medication, n (%)** | 465 (34.3%) | 215 (15.5%) | 680 (24.8%) |

**Supplemental Table 2. Baseline Characteristics of the UK Biobank Cohort**

|  | **Prevalent CAD**  **(N=2568)** | **No Prevalent CAD**  **(N=50450)** | **Overall**  **(N=53018)** |
| --- | --- | --- | --- |
| **Age (years), mean (SD)** | 62.4 (5.90) | 56.5 (8.21) | 56.8 (8.21) |
| **Self-Reported Race, n (%)** |  |  |  |
| White | 2436 (94.9%) | 47003 (93.2%) | 49439 (93.2%) |
| Other | 132 (5.1%) | 3447 (6.8%) | 3579 (6.8%) |
| **Female sex, n (%)** | 690 (26.9%) | 27892 (55.3%) | 28582 (53.9%) |
| **Diabetes, n (%)** | 565 (22.0%) | 2873 (5.7%) | 3438 (6.5%) |
| **Hypertension, n (%)** | 2158 (84.0%) | 26539 (52.6%) | 28697 (54.1%) |
| **LDL-C (mmol/L), mean (SD)** | 2.78 (0.780) | 3.56 (0.844) | 3.53 (0.858) |
| **BMI (kg/m^2^), mean (SD)** | 29.4 (4.89) | 27.4 (4.77) | 27.5 (4.79) |
| **Smoker, n (%)** | 1644 (64.0%) | 22446 (44.5%) | 24090 (45.4%) |
| **Cholesterol Medication, n (%)** | 520 (20.2%) | 3314 (6.6%) | 3834 (7.2%) |

**Supplemental Table 3. UK Biobank ICD codes for CAD**

| **Code Type** | **Code** | **Description** |
| --- | --- | --- |
| ICD9 | 4109 | Acute myocardial infarction |
| ICD9 | 4119 | Other acute and subacute forms of ischaemic heart disease |
| ICD9 | 4129 | Old myocardial infarction |
| ICD9 | 4140 | Coronary atherosclerosis |
| ICD9 | 4148 | Other specified forms of chronic ischaemic heart disease |
| ICD9 | 4149 | Chronic ischaemic heart disease, unspecified |
| ICD10 | I20.0 | Unstable angina |
| ICD10 | I21.0 | Acute transmural myocardial infarction of anterior wall |
| ICD10 | I21.1 | Acute transmural myocardial infarction of inferior wall |
| ICD10 | I21.2 | Acute transmural myocardial infarction of other sites |
| ICD10 | I21.3 | Acute transmural myocardial infarction of unspecified site |
| ICD10 | I21.4 | Acute subendocardial myocardial infarction |
| ICD10 | I21.9 | Acute myocardial infarction, unspecified |
| ICD10 | I22.0 | Subsequent myocardial infarction of anterior wall |
| ICD10 | I22.1 | Subsequent myocardial infarction of inferior wall |
| ICD10 | I22.8 | Subsequent myocardial infarction of other sites |
| ICD10 | I22.9 | Subsequent myocardial infarction of unspecified site |
| ICD10 | I23.0 | Haemopericardium as current complication following acute myocardial |
| ICD10 | I23.1 | Atrial septal defect as current complication following acute myocar |
| ICD10 | I23.2 | Ventricular septal defect as current complication following acute m |
| ICD10 | I23.3 | Rupture of cardiac wall without haemopericardium as current complic |
| ICD10 | I23.5 | Rupture of papillary muscle as current complication following acute |
| ICD10 | I23.6 | Thrombosis of atrium, auricular appendage, and ventricle as current |
| ICD10 | I23.8 | Other current complications following acute myocardial infarction |
| ICD10 | I24.0 | Coronary thrombosis not resulting in myocardial infarction |
| ICD10 | I24.1 | Dressler syndrome |
| ICD10 | I24.8 | Other forms of acute ischaemic heart disease |
| ICD10 | I24.9 | Acute ischaemic heart disease, unspecified |
| ICD10 | I25.0 | Atherosclerotic cardiovascular disease, so described |
| ICD10 | I25.1 | Atherosclerotic heart disease |
| ICD10 | I25.2 | Ischaemic cardiomyopathy |
| ICD10 | I25.6 | Silent myocardial ischaemia |
| ICD10 | I25.8 | Other forms of chronic ischaemic heart disease |
| ICD10 | I25.9 | Chronic ischaemic heart disease, unspecified |
| OPCS4 | K40.1 | Saphenous vein graft replacement of one coronary artery |
| OPCS4 | K40.2 | Saphenous vein graft replacement of two coronary arteries |
| OPCS4 | K40.3 | Saphenous vein graft replacement of three coronary arteries |
| OPCS4 | K40.4 | Saphenous vein graft replacement of four or more coronary arteries |
| OPCS4 | K40.8 | Other specified saphenous vein graft replacement of coronary artery |
| OPCS4 | K40.9 | Unspecified saphenous vein graft replacement of coronary artery |
| OPCS4 | K41.1 | Autograft replacement of one coronary artery NEC |
| OPCS4 | K41.2 | Autograft replacement of two coronary arteries NEC |
| OPCS4 | K41.3 | Autograft replacement of three coronary arteries NEC |
| OPCS4 | K41.4 | Autograft replacement of four or more coronary arteries NEC |
| OPCS4 | K41.8 | Other specified other autograft replacement of coronary artery |
| OPCS4 | K42.2 | Allograft replacement of two coronary arteries |
| OPCS4 | K42.4 | Allograft replacement of four or more coronary arteries |
| OPCS4 | K43.1 | Prosthetic replacement of one coronary artery |
| OPCS4 | K43.3 | Prosthetic replacement of three coronary arteries |
| OPCS4 | K43.9 | Unspecified prosthetic replacement of coronary artery |
| OPCS4 | K44.1 | Replacement of coronary arteries using multiple methods |
| OPCS4 | K44.2 | Revision of replacement of coronary artery |
| OPCS4 | K44.8 | Other specified other replacement of coronary artery |
| OPCS4 | K44.9 | Unspecified other replacement of coronary artery |
| OPCS4 | K45.1 | Double anastomosis of mammary arteries to coronary arteries |
| OPCS4 | K45.2 | Double anastomosis of thoracic arteries to coronary arteries NEC |
| OPCS4 | K45.3 | Anastomosis of mammary artery to left anterior descending coronary |
| OPCS4 | K45.4 | Anastomosis of mammary artery to coronary artery NEC |
| OPCS4 | K45.5 | Anastomosis of thoracic artery to coronary artery NEC |
| OPCS4 | K45.6 | Revision of connection of thoracic artery to coronary artery |
| OPCS4 | K45.8 | Other specified connection of thoracic artery to coronary artery |
| OPCS4 | K45.9 | Unspecified connection of thoracic artery to coronary artery |
| OPCS4 | K46.1 | Double implantation of mammary arteries into heart |
| OPCS4 | K46.2 | Double implantation of thoracic arteries into heart NEC |
| OPCS4 | K46.3 | Implantation of mammary artery into heart NEC |
| OPCS4 | K46.4 | Implantation of thoracic artery into heart NEC |
| OPCS4 | K46.9 | Unspecified other bypass of coronary artery |
| OPCS4 | K47.1 | Endarterectomy of coronary artery |
| OPCS4 | K47.2 | Repair of arteriovenous fistula of coronary artery |
| OPCS4 | K47.3 | Repair of aneurysm of coronary artery |
| OPCS4 | K47.8 | Other specified repair of coronary artery |
| OPCS4 | K48.2 | Transposition of coronary artery NEC |
| OPCS4 | K48.8 | Other specified other open operations on coronary artery |
| OPCS4 | K49.1 | Percutaneous transluminal balloon angioplasty of one coronary arter |
| OPCS4 | K49.2 | Percutaneous transluminal balloon angioplasty of multiple coronary |
| OPCS4 | K49.3 | Percutaneous transluminal balloon angioplasty of bypass graft of co |
| OPCS4 | K49.4 | Percutaneous transluminal cutting balloon angioplasty of coronary a |
| OPCS4 | K49.8 | Other specified transluminal balloon angioplasty of coronary artery |
| OPCS4 | K49.9 | Unspecified transluminal balloon angioplasty of coronary artery |

**Supplemental Table 4. Summary Statistics for Clinical Variables across Phenoclusters**

|  | **Overall** | **Cluster 1** | **Cluster 2** | **Cluster 3** |
| --- | --- | --- | --- | --- |
| n | 1724 | 329 | 834 | 561 |
| HRCP, n (%) | 822 (58.0) | 169 (63.5) | 461 (65.5) | 192 (43.0) |
| HRP without Obstructive CAD*, n (%) | 160 (10.8) | 25 (9.0) | 92 (13.5) | 43 (8.2) |
| HRP including Obstructive CAD**, n (%) | 267 (15.5) | 48 (14.6) | 165 (19.8) | 54 (9.6) |
| Obstructive CAD≥ 50%, n (%) | 238 (13.8) | 51 (15.5) | 152 (18.2) | 35 (6.2) |
| CAC Score > 400, n (%) | 204 (11.8) | 37 (11.2) | 132 (15.8) | 35 (6.2) |
| Leaman Score > 5, n (%) | 755 (43.8) | 160 (48.6) | 427 (51.2) | 168 (29.9) |
| HRP Positive Remodeling, n (%) | 245 (14.2) | 43 (13.1) | 149 (17.9) | 53 (9.4) |
| HRP Low Attenuation Plaque, n (%) | 80 (4.6) | 17 (5.2) | 53 (6.4) | 10 (1.8) |
| HRP Napkin Ring Sign, n (%) | 58 (3.4) | 10 (3.0) | 41 (4.9) | 7 (1.2) |
| Female sex, n (%) | 911 (52.8) | 150 (45.6) | 315 (37.8) | 446 (79.5) |
| Age, mean (SD) | 60.2 (8.0) | 57.6 (7.3) | 60.0 (8.2) | 62.2 (7.7) |
| Hypertension, n (%) | 1110 (64.4) | 220 (66.9) | 576 (69.1) | 314 (56.0) |
| Diabetes, n (%) | 343 (19.9) | 78 (23.7) | 220 (26.4) | 45 (8.0) |
| Dyslipidemia, n (%) | 1141 (66.2) | 253 (76.9) | 558 (66.9) | 330 (58.8) |
| Cerebrovascular Disease, n (%) | 61 (3.5) | 8 (2.4) | 36 (4.3) | 17 (3.0) |
| Peripheral Artery Disease, n (%) | 32 (1.9) | 3 (0.9) | 17 (2.0) | 12 (2.1) |
| Heart Failure, n (%) | 97 (5.6) | 20 (6.1) | 50 (6.0) | 27 (4.8) |
| Family History of premature CAD, n (%) | 580 (33.6) | 120 (36.5) | 260 (31.2) | 200 (35.7) |
| Body Mass Index, mean (SD) | 30.5 (5.8) | 31.4 (5.2) | 31.9 (5.9) | 28.0 (5.2) |
| Creatinine, mean (SD) | 0.9 (0.2) | 0.9 (0.2) | 0.9 (0.2) | 0.8 (0.2) |
| Smoking Status, n (%) | 904 (52.4) | 171 (52.0) | 460 (55.2) | 273 (48.7) |
| Metabolic Syndrome, n (%) | 637 (36.9) | 188 (57.1) | 386 (46.3) | 63 (11.2) |
| Minority Race, n (%) | 212 (12.3) | 30 (9.1) | 104 (12.5) | 78 (13.9) |
| LDL-C (mg/dL), mean (SD) | 121.0 (34.5) | 140.7 (40.3) | 112.7 (29.6) | 121.7 (32.6) |
| HDL-C (mg/dL), mean (SD) | 53.5 (13.5) | 46.9 (9.9) | 47.1 (8.0) | 66.7 (12.3) |
| Triglycerides, mean (SD) | 156.1 (111.5) | 303.3 (162.5) | 135.8 (52.6) | 100.0 (46.6) |
| Total Cholesterol (mg/dL), mean (SD) | 205.5 (42.2) | 240.1 (46.3) | 188.0 (33.1) | 211.3 (37.3) |
| Statin Usage, n (%) | 738 (42.8) | 127 (38.6) | 414 (49.6) | 197 (35.1) |
| Heart Rate, mean (SD) | 72.2 (11.9) | 74.7 (12.0) | 72.3 (12.0) | 70.8 (11.5) |
| Systolic Blood Pressure, mean (SD) | 130.9 (16.7) | 133.2 (17.2) | 131.4 (16.1) | 128.7 (17.1) |
| Diastolic Blood Pressure, mean (SD) | 78.6 (10.0) | 81.3 (9.8) | 79.1 (9.7) | 76.4 (9.9) |
| Lipoprotein(a), mean (SD) | 31.5 (36.6) | 26.7 (34.6) | 29.7 (35.6) | 37.0 (38.7) |
| NT-proBNP, mean (SD) | 128.1 (183.7) | 102.6 (156.9) | 112.4 (156.7) | 166.5 (225.1) |
| Cardiac Troponin I, mean (SD) | 3.6 (35.3) | 7.9 (80.1) | 2.8 (5.8) | 2.1 (3.4) |
| BNP, mean (SD) | 48.7 (60.9) | 37.9 (61.3) | 42.7 (48.8) | 63.9 (72.9) |
| Apolipoprotein B, mean (SD) | 97.5 (26.5) | 123.8 (29.3) | 91.2 (20.9) | 91.5 (22.3) |
| Apolipoprotein A1, mean (SD) | 149.0 (28.7) | 138.7 (24.2) | 136.7 (21.0) | 173.1 (26.0) |

*Presence of any HRP features but without oCAD in any coronary artery

**Anyone with HRP features regardless of whether oCAD is present in any coronary artery

Additional Variables used but not reported: Adiponectin, Alanine, ALT, Beta-2 Microglobulin Test, Branched-Chain Amino Acids, Cholesterol of HDL Particle, Citrate, Cystatin C, Depression, Free T3, Galectin-3 Test, Glucose, Glycated Hemoglobin, HDL Subspecies H1P, HDL Subspecies H2P, HDL Subspecies H3P, HDL Subspecies H4P, HDL Subspecies H5P, HDL Subspecies H6P, HDL Subspecies H7P, High-Density Lipoprotein Size, Insulin, Interleukin-6, Isoleucine, Kidney Injury Molecule-1, Large LDLP, Large TRLP, Leucine, Lipoprotein Insulin Resistance Index, Low-Density Lipoprotein Particles, Low-Density Lipoprotein Size, Matrix Metalloproteinase-9, Medium LDLP, Medium TRLP, Resistin, Small LDLP, Small TRLP, Total Homocysteine, Triglyceride-Rich Lipoprotein Particles, Triglyceride-Rich Lipoprotein Size, TRL Cholesterol, TRL Triglycerides, Uric Acid Test, Valine, Very Large TRLP, Very Small TRLP

**Supplemental Table 5.** **Clinical characteristics of PROMISE proteomics substudy and overall parent clinical trial**

|  | **PROMISE Substudy (N=1724)** | **PROMISE Clinical Trial (N=10003)** |
| --- | --- | --- |
| **Age (years), mean (SD)** | 60.2 (8.03) | 60.8 (8.30) |
| **Self-Reported Race, n (%)** |  |  |
| **Multi-Racial** | 19 (1.1%) | 95 (0.9%) |
| **White** | 1504 (87.2%) | 8371 (83.7%) |
| **Black or African American** | 149 (8.6%) | 1096 (11.0%) |
| **Asian** | 27 (1.6%) | 253 (2.5%) |
| **American Indian or Alaska Native** | 13 (0.8%) | 71 (0.7%) |
| **Native Hawaiian or Other Pacific Islander** | 4 (0.2%) | 30 (0.3%) |
| **Female sex, n (%)** | 911 (52.8%) | 5270 (52.7%) |
| **Diabetes, n (%)** | 343 (19.9%) | 2144 (21.4%) |
| **Hypertension, n (%)** | 1110 (64.4%) | 6501 (65.0%) |
| **LDLC, mean (SD)*** | 121 (34.7) | 121 (33.9) |
| **BMI (kg/m^2), mean (SD)** | 30.6 (5.88) | 30.5 (6.13) |
| **Statin, n (%)** | 738 (42.8%) | 4389 (43.9%) |
| **Smoker, n (%)** | 904 (52.4%) | 5104 (51.0%) |

**Supplemental Table 6**. **Results of Univariable Models for Association of Proteins with HRCP in the PROMISE cohort**

| **Olink ID** | **Protein** | **Odds Ratio [95% confidence interval]** | **P value** | **FDR adjusted P value** |
| --- | --- | --- | --- | --- |
| OID00457 | ACE2 | 1.77 [1.48 - 2.14] | 9.0E-10 | 8.5E-08 |
| OID01152 | ADGRG2 | 0.43 [0.33 - 0.57] | 1.2E-09 | 1.0E-07 |
| OID01156 | ALDH1A1 | 1.23 [1.07 - 1.42] | 4.0E-03 | 2.4E-02 |
| OID01413 | ANGPTL4 | 1.35 [1.15 - 1.60] | 3.7E-04 | 4.0E-03 |
| OID01151 | APLP1 | 0.75 [0.65 - 0.87] | 8.1E-05 | 1.2E-03 |
| OID00386 | BOC | 0.62 [0.45 - 0.85] | 3.0E-03 | 1.9E-02 |
| OID01477 | CA2 | 1.15 [1.04 - 1.28] | 6.0E-03 | 3.5E-02 |
| OID00505 | CCL11 | 1.43 [1.16 - 1.76] | 8.6E-04 | 7.4E-03 |
| OID00629 | CCL15 | 1.32 [1.11 - 1.57] | 1.9E-03 | 1.3E-02 |
| OID00654 | CCL16 | 1.30 [1.12 - 1.51] | 4.4E-04 | 4.3E-03 |
| OID00556 | CCL20 | 1.21 [1.09 - 1.34] | 3.5E-04 | 3.8E-03 |
| OID00539 | CCL28 | 1.35 [1.08 - 1.70] | 8.8E-03 | 4.8E-02 |
| OID00532 | CCL3 | 1.23 [1.06 - 1.44] | 9.0E-03 | 4.8E-02 |
| OID05579 | CCN5 | 1.53 [1.26 - 1.85] | 1.4E-05 | 3.3E-04 |
| OID01187 | CD1C | 0.63 [0.49 - 0.81] | 4.2E-04 | 4.3E-03 |
| OID05400 | CD22 | 0.80 [0.67 - 0.95] | 9.6E-03 | 5.0E-02 |
| OID01422 | CD300LG | 0.64 [0.52 - 0.77] | 6.2E-06 | 1.6E-04 |
| OID00476 | CDCP1 | 1.45 [1.22 - 1.72] | 3.0E-05 | 5.7E-04 |
| OID01189 | CDH2 | 1.43 [1.19 - 1.72] | 1.6E-04 | 2.0E-03 |
| OID05477 | CDHR2 | 1.36 [1.23 - 1.51] | 4.1E-09 | 2.9E-07 |
| OID01143 | CDHR5 | 0.69 [0.55 - 0.86] | 1.1E-03 | 9.2E-03 |
| OID01441 | CDON | 0.66 [0.52 - 0.84] | 8.5E-04 | 7.4E-03 |
| OID01450 | CGA | 0.73 [0.67 - 0.80] | 4.4E-11 | 8.3E-09 |
| OID05404 | CGB3 | 0.71 [0.64 - 0.78] | 1.5E-11 | 4.2E-09 |
| OID00633 | CHI3L1 | 1.30 [1.17 - 1.45] | 2.6E-06 | 7.3E-05 |
| OID01168 | CLUL1 | 0.69 [0.57 - 0.83] | 8.0E-05 | 1.2E-03 |
| OID00586 | CNTN1 | 0.65 [0.51 - 0.83] | 5.9E-04 | 5.7E-03 |
| OID00641 | COL1A1 | 0.64 [0.50 - 0.81] | 2.9E-04 | 3.3E-03 |
| OID01330 | COL4A1 | 1.30 [1.10 - 1.53] | 2.2E-03 | 1.5E-02 |
| OID00414 | CTRC | 0.81 [0.71 - 0.93] | 1.8E-03 | 1.3E-02 |
| OID00622 | CTSD | 1.55 [1.27 - 1.88] | 1.3E-05 | 3.0E-04 |
| OID00643 | CTSZ | 1.54 [1.22 - 1.94] | 2.8E-04 | 3.3E-03 |
| OID05466 | CXCL14 | 1.66 [1.38 - 2.00] | 9.7E-08 | 5.1E-06 |
| OID00490 | CXCL9 | 1.31 [1.16 - 1.49] | 3.4E-05 | 6.2E-04 |
| OID01213 | DNER | 0.64 [0.46 - 0.87] | 4.7E-03 | 2.8E-02 |
| OID00637 | EGFR | 0.60 [0.45 - 0.81] | 9.2E-04 | 7.7E-03 |
| OID05025 | ENPP2 | 0.77 [0.64 - 0.92] | 4.8E-03 | 2.8E-02 |
| OID01165 | ENTPD5 | 0.54 [0.39 - 0.73] | 1.1E-04 | 1.6E-03 |
| OID00610 | Ep-CAM | 0.82 [0.74 - 0.90] | 9.2E-05 | 1.3E-03 |
| OID00615 | FAS | 1.46 [1.17 - 1.83] | 9.1E-04 | 7.7E-03 |
| OID01126 | FCRL1 | 0.75 [0.63 - 0.89] | 1.1E-03 | 9.2E-03 |
| OID00512 | FGF-21 | 1.13 [1.05 - 1.22] | 2.2E-03 | 1.5E-02 |
| OID05457 | FLT3 | 0.70 [0.53 - 0.91] | 9.3E-03 | 4.8E-02 |
| OID01492 | FUT3/FUT5 | 1.63 [1.30 - 2.05] | 2.4E-05 | 4.7E-04 |
| OID00626 | Gal-4 | 1.23 [1.05 - 1.44] | 9.8E-03 | 5.0E-02 |
| OID01353 | GALNT2 | 1.47 [1.10 - 1.96] | 9.7E-03 | 5.0E-02 |
| OID05487 | GALNT7 | 0.61 [0.42 - 0.88] | 8.6E-03 | 4.7E-02 |
| OID01349 | GCNT1 | 1.41 [1.10 - 1.81] | 6.7E-03 | 3.8E-02 |
| OID00595 | GDF-15 | 2.10 [1.76 - 2.52] | 3.6E-16 | 2.0E-13 |
| OID00450 | GDF-2 | 0.78 [0.66 - 0.91] | 2.1E-03 | 1.5E-02 |
| OID05492 | GPA33 | 0.84 [0.78 - 0.91] | 7.8E-06 | 2.0E-04 |
| OID01463 | GUSB | 1.23 [1.09 - 1.39] | 7.7E-04 | 7.0E-03 |
| OID00522 | HGF | 1.41 [1.14 - 1.75] | 1.6E-03 | 1.1E-02 |
| OID05467 | HSPB6 | 1.54 [1.28 - 1.86] | 5.3E-06 | 1.4E-04 |
| OID00638 | IGFBP-7 | 1.37 [1.13 - 1.67] | 1.3E-03 | 1.0E-02 |
| OID01192 | IGFBPL1 | 1.47 [1.18 - 1.82] | 5.0E-04 | 4.8E-03 |
| OID00483 | IL-17C | 1.24 [1.09 - 1.42] | 1.4E-03 | 1.1E-02 |
| OID00640 | IL-18BP | 1.55 [1.24 - 1.95] | 1.6E-04 | 2.0E-03 |
| OID01385 | IL17RB | 1.25 [1.07 - 1.47] | 6.4E-03 | 3.6E-02 |
| OID00501 | IL18 | 1.24 [1.05 - 1.47] | 9.9E-03 | 5.0E-02 |
| OID00400 | IL1RL2 | 0.70 [0.56 - 0.87] | 1.7E-03 | 1.2E-02 |
| OID00471 | IL8 | 1.29 [1.13 - 1.49] | 3.4E-04 | 3.8E-03 |
| OID01414 | INHBC | 1.37 [1.10 - 1.70] | 5.1E-03 | 3.0E-02 |
| OID00426 | KIM1 | 1.42 [1.25 - 1.62] | 1.9E-07 | 7.7E-06 |
| OID00463 | LEP | 0.78 [0.70 - 0.86] | 1.1E-06 | 3.6E-05 |
| OID00446 | LPL | 0.56 [0.45 - 0.70] | 2.6E-07 | 9.1E-06 |
| OID00583 | LTBR | 1.40 [1.09 - 1.81] | 9.1E-03 | 4.8E-02 |
| OID00616 | MB | 1.31 [1.13 - 1.53] | 4.1E-04 | 4.3E-03 |
| OID01403 | MFGE8 | 1.31 [1.11 - 1.56] | 1.5E-03 | 1.1E-02 |
| OID05431 | MLN | 1.31 [1.18 - 1.45] | 2.0E-07 | 7.7E-06 |
| OID00510 | MMP-1 | 1.15 [1.05 - 1.27] | 3.4E-03 | 2.1E-02 |
| OID00527 | MMP-10 | 1.30 [1.11 - 1.53] | 1.5E-03 | 1.1E-02 |
| OID00644 | MMP-3 | 1.46 [1.27 - 1.68] | 1.3E-07 | 6.4E-06 |
| OID00568 | MMP-9 | 1.19 [1.04 - 1.35] | 9.3E-03 | 4.8E-02 |
| OID00456 | MMP12 | 1.51 [1.31 - 1.75] | 1.8E-08 | 1.0E-06 |
| OID01469 | MSMB | 1.24 [1.12 - 1.38] | 8.4E-05 | 1.3E-03 |
| OID01482 | OMD | 0.69 [0.59 - 0.82] | 1.8E-05 | 3.7E-04 |
| OID01341 | OMG | 0.75 [0.64 - 0.87] | 2.8E-04 | 3.3E-03 |
| OID00421 | PAPPA | 1.63 [1.29 - 2.07] | 4.4E-05 | 7.9E-04 |
| OID05494 | PCDH1 | 2.21 [1.37 - 3.57] | 1.2E-03 | 9.6E-03 |
| OID00518 | PD-L1 | 1.46 [1.16 - 1.84] | 1.6E-03 | 1.1E-02 |
| OID00384 | PGF | 1.77 [1.37 - 2.30] | 1.7E-05 | 3.6E-04 |
| OID00642 | PON3 | 0.68 [0.59 - 0.79] | 2.0E-07 | 7.7E-06 |
| OID01347 | PROK1 | 1.36 [1.20 - 1.53] | 8.0E-07 | 2.7E-05 |
| OID00447 | PRSS8 | 2.19 [1.68 - 2.88] | 1.1E-08 | 7.2E-07 |
| OID00608 | PSP-D | 1.34 [1.15 - 1.55] | 1.5E-04 | 2.0E-03 |
| OID05420 | PSPN | 1.33 [1.22 - 1.45] | 9.7E-11 | 1.4E-08 |
| OID01489 | RELT | 1.59 [1.25 - 2.02] | 1.6E-04 | 2.0E-03 |
| OID00423 | REN | 1.45 [1.29 - 1.63] | 2.6E-10 | 3.0E-08 |
| OID01193 | RTN4R | 1.63 [1.28 - 2.08] | 7.7E-05 | 1.2E-03 |
| OID05419 | S100A16 | 0.65 [0.53 - 0.81] | 9.5E-05 | 1.4E-03 |
| OID00408 | SCF | 0.61 [0.49 - 0.76] | 1.6E-05 | 3.5E-04 |
| OID01461 | SEMA7A | 1.34 [1.08 - 1.67] | 8.1E-03 | 4.5E-02 |
| OID01361 | SEZ6L2 | 0.53 [0.34 - 0.82] | 4.0E-03 | 2.4E-02 |
| OID00502 | SLAMF1 | 1.38 [1.09 - 1.75] | 7.8E-03 | 4.4E-02 |
| OID01391 | SLAMF8 | 1.38 [1.15 - 1.68] | 8.2E-04 | 7.4E-03 |
| OID01415 | SPINK1 | 1.29 [1.12 - 1.50] | 3.8E-04 | 4.0E-03 |
| OID00634 | ST2 | 1.42 [1.20 - 1.69] | 4.8E-05 | 8.1E-04 |
| OID00635 | t-PA | 1.32 [1.12 - 1.57] | 9.7E-04 | 8.1E-03 |
| OID01130 | TFF2 | 1.26 [1.10 - 1.44] | 6.9E-04 | 6.3E-03 |
| OID00462 | TGM2 | 1.26 [1.10 - 1.43] | 6.6E-04 | 6.2E-03 |
| OID00649 | TNF-R1 | 1.42 [1.13 - 1.80] | 3.1E-03 | 2.0E-02 |
| OID00561 | TNFB | 0.71 [0.57 - 0.88] | 2.3E-03 | 1.5E-02 |
| OID00563 | TNFRSF14 | 1.39 [1.12 - 1.73] | 3.1E-03 | 2.0E-02 |
| OID01490 | TPP1 | 1.36 [1.11 - 1.67] | 3.3E-03 | 2.1E-02 |
| OID00488 | TRAIL | 0.57 [0.42 - 0.78] | 4.4E-04 | 4.3E-03 |
| OID00396 | TRAIL-R2 | 1.35 [1.09 - 1.70] | 9.1E-03 | 4.8E-02 |
| OID00521 | TRANCE | 0.70 [0.59 - 0.83] | 4.7E-05 | 8.1E-04 |
| OID00555 | TWEAK | 0.62 [0.48 - 0.79] | 1.4E-04 | 1.9E-03 |
| OID00481 | uPA | 0.67 [0.52 - 0.87] | 2.9E-03 | 1.9E-02 |
| OID05408 | VMO1 | 1.34 [1.15 - 1.57] | 1.6E-04 | 2.0E-03 |
| OID00429 | VSIG2 | 1.32 [1.10 - 1.57] | 2.4E-03 | 1.6E-02 |
| OID01448 | VSIG4 | 1.34 [1.12 - 1.60] | 1.4E-03 | 1.0E-02 |
| OID01478 | XG | 0.64 [0.53 - 0.77] | 1.6E-06 | 4.9E-05 |

**Supplemental Table 7**. **Results of Multivariable Models for Association of Proteins with HRCP in the PROMISE cohort**

| **Olink ID** | **Protein** | **Odds Ratio [95% confidence interval]** | **P value** | **Full Name** |
| --- | --- | --- | --- | --- |
| OID00457 | ACE2 | 1.4 [1.14 - 1.73] | 1.6E-03 | Angiotensin-converting enzyme 2 |
| OID01152 | ADGRG2 | 0.55 [0.4 - 0.76] | 2.4E-04 | Adhesion G Protein-Coupled Receptor G2 |
| OID01151 | APLP1 | 0.8 [0.67 - 0.96] | 1.5E-02 | Amyloid-like protein 1 |
| OID00386 | BOC | 0.47 [0.32 - 0.69] | 1.1E-04 | Brother of CDO cell adhesion associated, oncogene regulated |
| OID00556 | CCL20 | 1.22 [1.08 - 1.38] | 1.8E-03 | C-C motif chemokine 20 |
| OID01422 | CD300LG | 0.68 [0.53 - 0.86] | 1.8E-03 | CD300 molecule like family member g |
| OID05477 | CDHR2 | 1.16 [1.01 - 1.32] | 3.1E-02 | Cadherin related family member 2 |
| OID01168 | CLUL1 | 0.79 [0.63 - 0.99] | 4.0E-02 | Clusterin Like 1 |
| OID00586 | CNTN1 | 0.70 [0.53 - 0.93] | 1.5E-02 | Contactin 1 |
| OID00641 | COL1A1 | 0.69 [0.52 - 0.91] | 9.1E-03 | Collagen, type I, alpha 1 |
| OID00414 | CTRC | 0.84 [0.73 - 0.98] | 2.7E-02 | Chymotrypsin-C |
| OID00622 | CTSD | 1.27 [1.01 - 1.61] | 4.5E-02 | Cathepsin D |
| OID01213 | DNER | 0.63 [0.43 - 0.92] | 1.8E-02 | Delta/Notch-like epidermal growth factor-related receptor |
| OID00637 | EGFR | 0.66 [0.47 - 0.93] | 1.9E-02 | Epidermal growth factor receptor |
| OID01165 | ENTPD5 | 0.61 [0.42 - 0.9] | 1.2E-02 | Ectonucleoside triphosphate diphosphohydrolase 5 |
| OID00610 | Ep-CAM | 0.87 [0.77 - 0.97] | 1.6E-02 | Epithelial cell adhesion molecule (CD326) |
| OID00512 | FGF-21 | 1.15 [1.05 - 1.26] | 3.6E-03 | Fibroblast growth factor 21 |
| OID05457 | FLT3 | 0.69 [0.5 - 0.94] | 1.9E-02 | Fms related receptor tyrosine kinase 3 |
| OID05487 | GALNT7 | 0.64 [0.41 - 0.99] | 4.5E-02 | N-acetylgalactosaminyltransferase 7 |
| OID00595 | GDF-15 | 1.35 [1.09 - 1.69] | 6.5E-03 | Growth/differentiation factor 15 |
| OID05492 | GPA33 | 0.9 [0.82 - 0.98] | 1.7E-02 | Glycoprotein A33 |
| OID00471 | IL8 | 1.22 [1.04 - 1.44] | 1.5E-02 | Interleukin 8 |
| OID01414 | INHBC | 1.41 [1.08 - 1.84] | 1.1E-02 | Inhibin beta C chain |
| OID00446 | LPL | 0.72 [0.56 - 0.93] | 1.3E-02 | Lipoprotein lipase |
| OID00616 | MB | 0.82 [0.68 - 0.99] | 3.7E-02 | Myoglobin |
| OID01403 | MFGE8 | 1.26 [1.03 - 1.54] | 2.4E-02 | Milk fat globule-EGF factor 8 protein (lactadherin) |
| OID05431 | MLN | 1.15 [1.01 - 1.3] | 3.3E-02 | Motilin |
| OID00456 | MMP12 | 1.28 [1.07 - 1.52] | 6.2E-03 | Matrix metallopeptidase 12 |
| OID01482 | OMD | 0.75 [0.61 - 0.91] | 3.8E-03 | Osteomodulin |
| OID01341 | OMG | 0.77 [0.64 - 0.94] | 8.2E-03 | Oligodendrocyte-myelin glycoprotein |
| OID00642 | PON3 | 0.74 [0.62 - 0.88] | 8.8E-04 | Paraoxonase 3 |
| OID00408 | SCF | 0.58 [0.44 - 0.76] | 1.2E-04 | Stem cell factor (KIT-ligand) |
| OID01415 | SPINK1 | 1.19 [1 - 1.41] | 4.9E-02 | Serine protease inhibitor Kazal-type 1 |
| OID00521 | TNFSF11 | 0.77 [0.63 - 0.95] | 1.5E-02 | TNF superfamily member 11 |
| OID00555 | TNFSF12 | 0.61 [0.46 - 0.82] | 9.1E-04 | TNF superfamily member 12 |
| OID00481 | uPA | 0.67 [0.49 - 0.91] | 1.0E-02 | Urokinase-type plasminogen activator |
| OID05408 | VMO1 | 1.23 [1.03 - 1.47] | 2.3E-02 | Vitelline membrane outer layer 1 homolog |

**Supplemental Table 8. Cox Time-to-MACE Models for Individual Proteins from the Proteomic Score in the PROMISE Cohort**

| **Protein** | **Univariable Hazard Ratio (95% CI)** | **Univariable P Value** | **FDR P adjusted value** | **Multivariable Hazard Ratio (95% CI)** | **Multivariable P Value** |
| --- | --- | --- | --- | --- | --- |
| ACE2 | 1.78 (1.29 - 2.45) | 4.07E-04 | 1.17E-02 | 1.83 (1.31 - 2.55) | 3.53E-04 |
| ADGRG2 | 0.55 (0.28 - 1.07) | 7.88E-02 | 1.94E-01 |  |  |
| APLP1 | 0.78 (0.57 - 1.08) | 1.40E-01 | 3.11E-01 |  |  |
| BOC | 0.93 (0.43 - 2.01) | 8.52E-01 | 8.75E-01 |  |  |
| CCL20 | 1.24 (1.01 - 1.53) | 4.15E-02 | 1.28E-01 |  |  |
| CD300LG | 1.01 (0.64 - 1.61) | 9.54E-01 | 9.54E-01 |  |  |
| CDHR2 | 1.39 (1.09 - 1.77) | 8.12E-03 | 4.29E-02 | 1.52 (1.16 - 1.98) | 2.30E-03 |
| CLUL1 | 0.88 (0.57 - 1.36) | 5.71E-01 | 6.98E-01 |  |  |
| CNTN1 | 0.81 (0.45 - 1.45) | 4.80E-01 | 6.42E-01 |  |  |
| COL1A1 | 0.93 (0.52 - 1.66) | 8.06E-01 | 8.52E-01 |  |  |
| CTRC | 0.78 (0.59 - 1.03) | 7.81E-02 | 1.94E-01 |  |  |
| CTSD | 1.83 (1.20 - 2.79) | 4.66E-03 | 2.87E-02 | 1.87 (1.19 - 2.93) | 6.58E-03 |
| DNER | 1.28 (0.59 - 2.77) | 5.28E-01 | 6.73E-01 |  |  |
| EGFR | 0.69 (0.34 - 1.39) | 3.01E-01 | 5.07E-01 |  |  |
| ENTPD5 | 0.74 (0.34 - 1.60) | 4.47E-01 | 6.36E-01 |  |  |
| Ep-CAM | 0.77 (0.60 - 0.99) | 4.52E-02 | 1.29E-01 |  |  |
| FGF-21 | 1.12 (0.94 - 1.35) | 2.03E-01 | 3.95E-01 |  |  |
| FLT3 | 0.79 (0.40 - 1.55) | 4.86E-01 | 6.42E-01 |  |  |
| GALNT7 | 0.35 (0.15 - 0.81) | 1.39E-02 | 5.72E-02 |  |  |
| GDF-15 | 1.71 (1.23 - 2.36) | 1.26E-03 | 1.17E-02 | 1.68 (1.12 - 2.50) | 1.16E-02 |
| GPA33 | 0.82 (0.68 - 0.98) | 3.25E-02 | 1.09E-01 |  |  |
| IL8 | 1.40 (1.08 - 1.82) | 1.23E-02 | 5.70E-02 |  |  |
| INHBC | 1.11 (0.66 - 1.86) | 6.87E-01 | 7.71E-01 |  |  |
| LPL | 0.56 (0.40 - 0.79) | 9.98E-04 | 1.17E-02 | 0.54 (0.37 - 0.77) | 7.89E-04 |
| MB | 0.93 (0.65 - 1.34) | 7.13E-01 | 7.76E-01 |  |  |
| MFGE8 | 0.89 (0.59 - 1.34) | 5.85E-01 | 6.98E-01 |  |  |
| MLN | 1.11 (0.88 - 1.41) | 3.84E-01 | 5.93E-01 |  |  |
| MMP12 | 1.74 (1.26 - 2.40) | 8.33E-04 | 1.17E-02 | 1.53 (1.10 - 2.15) | 1.27E-02 |
| OMD | 0.78 (0.52 - 1.18) | 2.40E-01 | 4.43E-01 |  |  |
| OMG | 0.91 (0.62 - 1.33) | 6.20E-01 | 7.17E-01 |  |  |
| PON3 | 0.71 (0.53 - 0.95) | 2.34E-02 | 8.66E-02 |  |  |
| SCF | 0.51 (0.33 - 0.79) | 2.27E-03 | 1.68E-02 | 0.54 (0.35 - 0.83) | 4.93E-03 |
| SPINK1 | 1.15 (0.84 - 1.58) | 3.82E-01 | 5.93E-01 |  |  |
| TRANCE | 0.85 (0.57 - 1.28) | 4.41E-01 | 6.36E-01 |  |  |
| TWEAK | 0.65 (0.37 - 1.16) | 1.43E-01 | 3.11E-01 |  |  |
| uPA | 1.38 (0.75 - 2.54) | 2.96E-01 | 5.07E-01 |  |  |
| VMO1 | 1.31 (0.88 - 1.95) | 1.85E-01 | 3.80E-01 |  |  |

**Supplemental Table 9. Gene Set Enrichment Analyses for HRCP (Based on Univariable HRCP Models, Top 10 Hallmark Pathways Displayed)**

|  | **Hallmark Pathway** | **Enrichment Score** | **Nominal p-value** | **FDR adjusted p-value** |
| --- | --- | --- | --- | --- |
| **1** | ADIPOGENESIS | 0.16 | 0.075 | 0.95 |
| **2** | ESTROGEN_RESPONSE_LATE | 0.14 | 0.096 | 0.95 |
| **3** | PI3K_AKT_MTOR_SIGNALING | 0.11 | 0.15 | 0.95 |
| **4** | EPITHELIAL_MESENCHYMAL_TRANSITION | 0.08 | 0.21 | 0.95 |
| **5** | ESTROGEN_RESPONSE_EARLY | 0.08 | 0.27 | 0.95 |
| **6** | COAGULATION | 0.07 | 0.29 | 0.95 |
| **7** | CHOLESTEROL_HOMEOSTASIS | 0.07 | 0.30 | 0.95 |
| **8** | PROTEIN_SECRETION | 0.06 | 0.33 | 0.95 |
| **9** | IL2_STAT5_SIGNALING | 0.06 | 0.34 | 0.95 |
| **10** | HYPOXIA | 0.06 | 0.37 | 0.95 |

**Supplemental Table 10. Gene Set Enrichment Analyses for HRCP (Based on Univariable HRCP Models, Top 10 KEGG Pathways Displayed)**

|  | **KEGG Pathway** | **Enrichment Score** | **Nominal p-value** | **FDR adjusted p-value** |
| --- | --- | --- | --- | --- |
| **1** | Renin-angiotensin system | *0.46* | *0.001* | *0.17* |
| **2** | Neuroactive ligand-receptor interaction | *0.41* | *0.002* | *0.17* |
| **3** | Cytokine-cytokine receptor interaction | *0.28* | *0.024* | *1.0* |
| **4** | GnRH signaling pathway | *0.27* | *0.039* | *1.0* |
| **5** | Prolactin signaling pathway | *0.22* | *0.040* | *1.0* |
| **6** | Parathyroid hormone synthesis, secretion and action | *0.24* | *0.045* | *1.0* |
| **7** | Ovarian steroidogenesis | *0.20* | *0.054* | *1.0* |
| **8** | Phospholipase D signaling pathway | *0.20* | *0.060* | *1.0* |
| **9** | Glycosphingolipid biosynthesis - lacto and neolacto series | *0.15* | *0.080* | *1.0* |
| **10** | Regulation of lipolysis in adipocytes | *0.16* | *0.093* | *1.0* |

**Supplemental Table 11. Protein Levels Differences Between Phenoclusters in PROMISE.**

| **Olink ID** | **Protein** | **Cluster 1  Mean (SD)** | **Cluster 2 Mean (SD)** | **Cluster 3 Mean (SD)** | **ANOVA P Value** | **FDR** |
| --- | --- | --- | --- | --- | --- | --- |
| OID00457 | ACE2 | 3.58 (0.69) | 3.4 (0.62) | 3.21 (0.58) | 2.9E-17 | 8.1E-17 |
| OID01152 | ADGRG2 | 2.19 (0.4) | 2.15 (0.39) | 2.35 (0.39) | 1.1E-18 | 3.3E-18 |
| OID01151 | APLP1 | 6.69 (0.75) | 6.71 (0.77) | 7.11 (0.71) | 6.8E-24 | 3.1E-23 |
| OID00386 | BOC | 3.63 (0.33) | 3.61 (0.33) | 3.65 (0.34) | 1.3E-01 | 1.4E-01 |
| OID00556 | CCL20 | 7.69 (1.04) | 7.48 (1.09) | 7.31 (1.05) | 2.1E-06 | 3.5E-06 |
| OID01422 | CD300LG | 3.39 (0.54) | 3.41 (0.52) | 3.73 (0.55) | 2.8E-31 | 1.5E-30 |
| OID05477 | CDHR2 | 3.10 (1) | 2.89 (0.93) | 2.09 (1.06) | 2.6E-60 | 9.8E-59 |
| OID01168 | CLUL1 | 3.84 (0.56) | 3.84 (0.56) | 4.19 (0.55) | 3.1E-33 | 1.9E-32 |
| OID00586 | CNTN1 | 4.39 (0.41) | 4.35 (0.44) | 4.47 (0.46) | 6.0E-06 | 9.2E-06 |
| OID00641 | COL1A1 | 2.36 (0.41) | 2.37 (0.43) | 2.43 (0.48) | 2.1E-02 | 2.5E-02 |
| OID00414 | CTRC | 10.84 (0.8) | 10.68 (0.84) | 10.77 (0.81) | 7.8E-03 | 1.0E-02 |
| OID00622 | CTSD | 4.05 (0.58) | 3.87 (0.55) | 3.67 (0.53) | 5.3E-23 | 2.0E-22 |
| OID01213 | DNER | 8.71 (0.33) | 8.69 (0.35) | 8.74 (0.33) | 1.8E-02 | 2.2E-02 |
| OID00637 | EGFR | 3.05 (0.36) | 2.98 (0.36) | 3.02 (0.35) | 1.7E-03 | 2.4E-03 |
| OID01165 | ENTPD5 | 1.63 (0.3) | 1.69 (0.33) | 1.81 (0.35) | 1.2E-16 | 3.2E-16 |
| OID00610 | Ep-CAM | 5.66 (1.06) | 5.61 (0.97) | 6.06 (1.11) | 2.2E-15 | 5.1E-15 |
| OID00512 | FGF-21 | 6.35 (1.33) | 5.81 (1.35) | 5.55 (1.37) | 3.0E-16 | 7.3E-16 |
| OID05457 | FLT3 | 0.86 (0.48) | 0.88 (0.4) | 0.87 (0.37) | 6.7E-01 | 6.7E-01 |
| OID05487 | GALNT7 | 4.48 (0.27) | 4.44 (0.29) | 4.46 (0.29) | 1.4E-01 | 1.5E-01 |
| OID00595 | GDF-15 | 5.25 (0.61) | 5.26 (0.68) | 5.16 (0.66) | 2.1E-02 | 2.5E-02 |
| OID05492 | GPA33 | 6.19 (1.42) | 6.11 (1.37) | 6.69 (1.47) | 2.4E-13 | 4.8E-13 |
| OID00471 | IL8 | 5.11 (0.78) | 5.08 (0.82) | 4.98 (0.75) | 3.2E-02 | 3.7E-02 |
| OID01414 | INHBC | 2.93 (0.47) | 2.78 (0.48) | 2.63 (0.5) | 4.5E-19 | 1.5E-18 |
| OID00446 | LPL | 9.79 (0.49) | 9.86 (0.55) | 10.21 (0.43) | 3.1E-42 | 2.8E-41 |
| OID00616 | MB | 6.31 (0.75) | 6.46 (0.74) | 6.17 (0.61) | 1.1E-13 | 2.5E-13 |
| OID01403 | MFGE8 | 4.31 (0.69) | 3.77 (0.59) | 3.71 (0.57) | 7.9E-49 | 1.1E-47 |
| OID05431 | MLN | 3.3 (1.03) | 3.01 (1.08) | 2.97 (1.06) | 1.7E-05 | 2.6E-05 |
| OID00456 | MMP12 | 7.68 (0.77) | 7.64 (0.77) | 7.57 (0.75) | 9.8E-02 | 1.1E-01 |
| OID01482 | OMD | 2.81 (0.65) | 2.85 (0.62) | 3.05 (0.67) | 1.4E-09 | 2.5E-09 |
| OID01341 | OMG | 2.31 (0.68) | 2.33 (0.66) | 2.55 (0.67) | 2.1E-10 | 4.2E-10 |
| OID00642 | PON3 | 5.21 (0.73) | 5.31 (0.74) | 5.85 (0.71) | 9.0E-49 | 1.1E-47 |
| OID00408 | SCF | 8.98 (0.54) | 9.23 (0.47) | 9.39 (0.42) | 1.5E-34 | 1.1E-33 |
| OID01415 | SPINK1 | 3.48 (0.74) | 3.46 (0.76) | 3.53 (0.8) | 2.6E-01 | 2.7E-01 |
| OID00521 | TRANCE | 4.39 (0.59) | 4.28 (0.6) | 4.12 (0.67) | 7.9E-10 | 1.5E-09 |
| OID00555 | TWEAK | 8.74 (0.43) | 8.76 (0.43) | 8.87 (0.43) | 3.1E-06 | 5.0E-06 |
| OID00481 | uPA | 9.85 (0.37) | 9.81 (0.42) | 9.89 (0.44) | 1.0E-03 | 1.5E-03 |
| OID05408 | VMO1 | 3.48 (0.7) | 3.14 (0.62) | 3.01 (0.7) | 5.3E-23 | 2.0E-22 |

**Supplemental Table 12: Reclassification of HRCP status after addition of the protein score to the clinical model in PROMISE**

| *Reclassification among participants without HRCP (N=595)* | | | | | |
| --- | --- | --- | --- | --- | --- |
|  |  | **Clinical Model +**  **Protein Score** | | |  |
|  |  | Low | Medium | High | % reclassified |
| **Clinical Model only** | Low | 143 | 30 | 1 | 18 |
|  | Medium | 69 | 235 | 16 | 27 |
|  | High | 0 | 32 | 69 | 32 |
| *Reclassification among participants with HRCP (N=822)* | | | | | |
|  |  | **Clinical Model +**  **Protein Score** | | |  |
|  |  | Low | Medium | High | % reclassified |
| **Clinical Model only** | Low | 55 | 15 | 1 | 23 |
|  | Medium | 18 | 203 | 76 | 32 |
|  | High | 0 | 43 | 411 | 9 |

| Reclassification among participants without HRCP (N=595) | | | | | |
| --- | --- | --- | --- | --- | --- |
|  |  | Clinical + Protein Score Model | | |  |
|  | Initial Model | Low | Medium | High | % reclassified |
| Clinical Model | Low | 143 | 30 | 1 | 18 |
|  | Medium | 69 | 235 | 16 | 27 |
|  | High | 0 | 32 | 69 | 32 |
| Reclassification among participants with HRCP (N=822) | | | | | |
|  |  | Clinical + Protein Score Model | | |  |
|  | Initial Model | Low | Medium | High | % reclassified |
| Clinical Model | Low | 55 | 15 | 1 | 23 |
|  | Medium | 18 | 203 | 76 | 32 |
|  | High | 0 | 43 | 411 | 9 |

Footnote: Rows represent risk categories from the clinical-only model (age, race, sex, diabetes, hypertension, LDL-C, BMI, statin use, smoking); columns represent risk categories after adding the elastic net-derived protein score. Risk categories are defined by tertiles of predicted probability. The “% reclassified” column shows the proportion of participants in each clinical-model-only category who were assigned to a different category after adding the protein score. A positive NRI indicates that, on balance, the protein score shifted participants without HRCP toward lower risk and those with HRCP toward higher risk; here it reflects a net improvement in correct classification, with the IDI showing a corresponding gain in discrimination.

Overall categorical NRI = 0.13 (95% CI: 0.08–0.18; p<0.001); IDI = 0.04 (95% CI: 0.03–0.05; p<0.001).

**Supplemental Table 13: Dan-NICAD validation protein association with HRCP**

| **Olink ID** | **Protein** | **Odds Ratio Univariable** | **P Value Univariable** | **FDR adjusted P Value Univariable** | **Odds Ratio Multivariable** | **P Value Multivariable** |
| --- | --- | --- | --- | --- | --- | --- |
| OID00457 | ACE2 | 1.43 (1.32, 1.55) | 2.9E-18 | 1.8E-17 | 1.08 (0.98, 1.19) | 1.1E-01 |
| OID01152 | ADGRG2 | 0.77 (0.71, 0.83) | 8.8E-11 | 4.1E-10 | 0.97 (0.88, 1.07) | 5.3E-01 |
| OID01151 | APLP1 | 0.87 (0.81, 0.94) | 3.7E-04 | 6.0E-04 | 0.95 (0.86, 1.04) | 2.9E-01 |
| OID00386 | BOC | 0.93 (0.86, 1) | 6.5E-02 | 7.6E-02 | 1.01 (0.93, 1.11) | 7.7E-01 |
| OID00556 | CCL20 | 1.09 (1.01, 1.17) | 2.6E-02 | 3.2E-02 | 1.03 (0.94, 1.13) | 5.3E-01 |
| OID01422 | CD300LG | 0.88 (0.82, 0.95) | 1.2E-03 | 1.7E-03 | 0.92 (0.84, 1.01) | 7.7E-02 |
| OID05477 | CDHR2 | 1.43 (1.32, 1.55) | 2.8E-19 | 2.6E-18 | 0.98 (0.88, 1.09) | 7.4E-01 |
| OID01168 | CLUL1 | 0.87 (0.81, 0.94) | 4.1E-04 | 6.3E-04 | 0.88 (0.8, 0.97) | 1.1E-02 |
| OID00586 | CNTN1 | 0.8 (0.74, 0.87) | 3.5E-08 | 1.2E-07 | 0.89 (0.8, 0.98) | 1.6E-02 |
| OID00641 | COL1A1 | 0.82 (0.76, 0.89) | 6.0E-07 | 1.7E-06 | 0.94 (0.86, 1.03) | 2.0E-01 |
| OID00414 | CTRC | 0.93 (0.86, 1) | 5.4E-02 | 6.4E-02 | 1.01 (0.93, 1.11) | 7.6E-01 |
| OID00622 | CTSD | 1.46 (1.33, 1.59) | 1.7E-16 | 8.8E-16 | 1.16 (1.05, 1.27) | 2.7E-03 |
| OID01213 | DNER | 0.98 (0.91 1.06) | 6.8E-01 | 6.8E-01 | 1.04 (0.95, 1.15) | 3.6E-01 |
| OID00637 | EGFR | 0.77 (0.71, 0.83) | 5.6E-10 | 2.3E-09 | 0.89 (0.81, 0.98) | 2.0E-02 |
| OID01165 | ENTPD5 | 0.96 (0.89, 1.04) | 3.2E-01 | 3.4E-01 | 0.96 (0.88, 1.05) | 3.5E-01 |
| OID00610 | Ep-CAM | 0.96 (0.89, 1.03) | 2.8E-01 | 3.1E-01 | 1.06 (0.97, 1.16) | 1.9E-01 |
| OID00512 | FGF-21 | 1.25 (1.15, 1.35) | 1.7E-08 | 6.3E-08 | 1.07 (0.98, 1.17) | 1.4E-01 |
| OID05457 | FLT3 | 0.82 (0.76, 0.89) | 1.1E-06 | 2.5E-06 | 0.96 (0.88, 1.06) | 4.3E-01 |
| OID05487 | GALNT7 | 0.91 (0.85, 0.99) | 2.1E-02 | 2.6E-02 | 0.98 (0.9, 1.08) | 7.2E-01 |
| OID00595 | GDF-15 | 1.95 (1.78, 2.13) | 1.7E-47 | 6.2E-46 | 1.16 (1.04, 1.29) | 8.9E-03 |
| OID05492 | GPA33 | 0.94 (0.87, 1.01) | 8.5E-02 | 9.6E-02 | 1.05 (0.96, 1.14) | 3.3E-01 |
| OID00471 | IL8 | 1.19 (1.1, 1.29) | 8.7E-06 | 1.6E-05 | 1.03 (0.94, 1.13) | 4.9E-01 |
| OID01414 | INHBC | 1.09 (1.01, 1.18) | 2.0E-02 | 2.6E-02 | 0.96 (0.87, 1.06) | 4.1E-01 |
| OID00446 | LPL | 0.83 (0.77, 0.89) | 1.0E-06 | 2.5E-06 | 1.01 (0.91, 1.11) | 8.6E-01 |
| OID00616 | MB | 1.44 (1.33, 1.56) | 9.8E-19 | 7.2E-18 | 0.99 (0.9, 1.1) | 9.2E-01 |
| OID01403 | MFGE8 | 1.19 (1.11, 1.29) | 5.4E-06 | 1.2E-05 | 1.12 (1.02, 1.22) | 1.5E-02 |
| OID05431 | MLN | 1.47 (1.36, 1.59) | 1.5E-21 | 1.9E-20 | 1.1 (1, 1.21) | 5.7E-02 |
| OID00456 | MMP12 | 1.74 (1.6, 1.9) | 3.1E-38 | 5.7E-37 | 1.28 (1.16, 1.41) | 1.4E-06 |
| OID01482 | OMD | 0.89 (0.83, 0.96) | 2.5E-03 | 3.4E-03 | 0.95 (0.87, 1.04) | 2.7E-01 |
| OID01341 | OMG | 0.84 (0.78, 0.91) | 7.1E-06 | 1.5E-05 | 0.93 (0.85, 1.02) | 1.2E-01 |
| OID00642 | PON3 | 0.82 (0.75, 0.89) | 7.9E-06 | 1.5E-05 | 0.95 (0.87, 1.04) | 2.9E-01 |
| OID00408 | SCF | 0.88 (0.81, 0.95) | 7.7E-04 | 1.1E-03 | 0.83 (0.76, 0.92) | 1.3E-04 |
| OID01415 | SPINK1 | 1.22 (1.13, 1.31) | 6.8E-07 | 1.8E-06 | 1.12 (1.02, 1.23) | 1.8E-02 |
| OID00521 | TRANCE | 0.81 (0.75, 0.88) | 8.0E-08 | 2.5E-07 | 0.91 (0.83, 1) | 5.0E-02 |
| OID00555 | TWEAK | 0.87 (0.81, 0.94) | 2.8E-04 | 4.7E-04 | 0.92 (0.84, 1) | 5.2E-02 |
| OID00481 | uPA | 0.98 (0.91, 1.06) | 6.6E-01 | 6.8E-01 | 0.98 (0.9, 1.07) | 6.5E-01 |
| OID05408 | VMO1 | 1.18 (1.09, 1.27) | 3.5E-05 | 6.2E-05 | 1.04 (0.95, 1.14) | 3.7E-01 |

**Supplemental Table 14: UK Biobank validation protein association with prevalent CAD**

| **Protein** | **Univariable Odds Ratio (95% CI)** | **Univariable P Value** | **Univariable FDR Adjusted P Value** | **Multivariable Odds Ratio (95% CI)** | **Multivariable P Value** | **Multivariable FDR Adjusted P Value** |
| --- | --- | --- | --- | --- | --- | --- |
| ACE2 | 2.29 (2.18 - 2.41) | 1.74E-240 | 2.14E-239 | 1.43 (1.34 - 1.52) | 1.28E-29 | 1.57E-28 |
| ADGRG2 | 0.16 (0.14 - 0.18) | 6.09E-196 | 5.63E-195 | 0.60 (0.52 - 0.69) | 3.57E-13 | 1.65E-12 |
| APLP1 | 0.78 (0.73 - 0.83) | 3.32E-13 | 4.24E-13 | 1.12 (1.04 - 1.21) | 1.96E-03 | 3.16E-03 |
| BOC | 0.86 (0.74 - 1.00) | 4.74E-02 | 4.74E-02 | 1.03 (0.87 - 1.22) | 7.18E-01 | 7.59E-01 |
| CCL20 | 1.32 (1.28 - 1.37) | 1.07E-65 | 3.04E-65 | 1.17 (1.12 - 1.21) | 2.07E-15 | 1.27E-14 |
| CD300LG | 0.59 (0.54 - 0.65) | 2.55E-28 | 3.93E-28 | 0.71 (0.64 - 0.78) | 4.39E-12 | 1.80E-11 |
| CDHR2 | 1.55 (1.49 - 1.62) | 1.85E-95 | 6.84E-95 | 0.94 (0.89 - 0.99) | 2.92E-02 | 4.50E-02 |
| CLUL1 | 0.65 (0.60 - 0.70) | 4.02E-29 | 6.76E-29 | 0.96 (0.88 - 1.05) | 3.85E-01 | 4.74E-01 |
| CNTN1 | 0.44 (0.39 - 0.50) | 6.36E-41 | 1.38E-40 | 0.78 (0.68 - 0.89) | 3.05E-04 | 5.64E-04 |
| COL1A1 | 0.72 (0.62 - 0.83) | 4.96E-06 | 5.40E-06 | 0.95 (0.82 - 1.11) | 5.51E-01 | 6.37E-01 |
| CTRC | 0.82 (0.78 - 0.87) | 2.39E-12 | 2.94E-12 | 1.01 (0.96 - 1.07) | 7.07E-01 | 7.59E-01 |
| CTSD | 2.09 (1.96 - 2.24) | 3.38E-99 | 1.39E-98 | 1.17 (1.07 - 1.28) | 4.03E-04 | 6.86E-04 |
| DNER | 0.41 (0.36 - 0.46) | 5.96E-40 | 1.16E-39 | 0.90 (0.78 - 1.04) | 1.67E-01 | 2.29E-01 |
| EGFR | 0.05 (0.04 - 0.06) | 2.74E-187 | 2.02E-186 | 0.31 (0.24 - 0.39) | 2.65E-23 | 2.45E-22 |
| ENTPD5 | 0.54 (0.46 - 0.63) | 1.14E-13 | 1.56E-13 | 0.90 (0.75 - 1.07) | 2.42E-01 | 3.20E-01 |
| Ep-CAM | 0.91 (0.88 - 0.95) | 1.81E-06 | 2.09E-06 | 1.00 (0.96 - 1.04) | 9.26E-01 | 9.52E-01 |
| FGF-21 | 1.25 (1.21 - 1.28) | 3.55E-60 | 8.20E-60 | 1.08 (1.04 - 1.11) | 1.85E-06 | 4.27E-06 |
| FLT3 | 0.42 (0.37 - 0.47) | 1.20E-40 | 2.47E-40 | 0.75 (0.66 - 0.86) | 1.89E-05 | 3.68E-05 |
| GALNT7 | 0.76 (0.64 - 0.91) | 1.95E-03 | 2.00E-03 | 1.00 (0.84 - 1.21) | 9.66E-01 | 9.66E-01 |
| GDF-15 | 3.23 (3.06 - 3.40) | 0.00E+00 | 0.00E+00 | 1.61 (1.50 - 1.73) | 1.49E-39 | 2.76E-38 |
| GPA33 | 0.93 (0.90 - 0.95) | 2.43E-08 | 2.90E-08 | 1.01 (0.99 - 1.04) | 3.52E-01 | 4.49E-01 |
| IL8 | 1.22 (1.17 - 1.28) | 2.28E-18 | 3.37E-18 | 1.04 (0.99 - 1.10) | 1.08E-01 | 1.60E-01 |
| INHBC | 2.17 (1.99 - 2.36) | 3.38E-71 | 1.04E-70 | 1.34 (1.21 - 1.47) | 7.39E-09 | 2.40E-08 |
| LPL | 0.57 (0.53 - 0.60) | 1.96E-74 | 6.61E-74 | 0.83 (0.76 - 0.89) | 1.59E-06 | 3.92E-06 |
| MB | 1.99 (1.89 - 2.09) | 2.33E-164 | 1.44E-163 | 1.16 (1.08 - 1.23) | 1.18E-05 | 2.44E-05 |
| MFGE8 | 1.67 (1.55 - 1.80) | 6.29E-40 | 1.16E-39 | 1.25 (1.15 - 1.36) | 1.89E-07 | 4.99E-07 |
| MLN | 1.38 (1.33 - 1.43) | 4.20E-61 | 1.11E-60 | 1.08 (1.04 - 1.13) | 4.08E-04 | 6.86E-04 |
| MMP12 | 2.77 (2.62 - 2.92) | 8.79E-304 | 1.63E-302 | 1.77 (1.66 - 1.88) | 5.16E-71 | 1.91E-69 |
| OMD | 0.75 (0.70 - 0.81) | 2.16E-13 | 2.86E-13 | 1.02 (0.94 - 1.12) | 6.05E-01 | 6.79E-01 |
| OMG | 0.80 (0.76 - 0.84) | 8.95E-17 | 1.27E-16 | 1.02 (0.96 - 1.08) | 5.15E-01 | 6.14E-01 |
| PON3 | 0.28 (0.26 - 0.31) | 3.15E-159 | 1.66E-158 | 0.61 (0.55 - 0.68) | 2.68E-19 | 1.98E-18 |
| SCF | 0.80 (0.73 - 0.88) | 2.42E-06 | 2.71E-06 | 0.77 (0.70 - 0.85) | 1.06E-07 | 3.02E-07 |
| SPINK1 | 2.18 (2.04 - 2.33) | 2.57E-116 | 1.19E-115 | 1.35 (1.25 - 1.46) | 6.85E-14 | 3.62E-13 |
| TRANCE | 0.62 (0.58 - 0.65) | 2.25E-60 | 5.55E-60 | 0.83 (0.77 - 0.88) | 5.15E-09 | 1.91E-08 |
| TWEAK | 0.50 (0.44 - 0.56) | 1.18E-28 | 1.89E-28 | 0.91 (0.79 - 1.04) | 1.63E-01 | 2.29E-01 |
| uPA | 1.28 (1.15 - 1.43) | 9.12E-06 | 9.64E-06 | 1.32 (1.17 - 1.48) | 4.63E-06 | 1.01E-05 |
| VMO1 | 1.44 (1.36 - 1.52) | 8.78E-36 | 1.55E-35 | 1.19 (1.12 - 1.26) | 7.79E-09 | 2.40E-08 |

**Supplemental Table 15: UK Biobank Time-to-Incident CAD Event Cox Models**

| **Protein** | **Univariable Hazard Ratio (95% CI)** | **Univariable P Value** | **FDR P adjusted value** | **Multivariable Hazard Ratio (95% CI)** | **Multivariable P Value** |
| --- | --- | --- | --- | --- | --- |
| ACE2***** | 2.05 (1.99 - 2.11) | 1.37E-152 | 1.37E-152 | 1.15 (1.09 - 1.20) | 1.87E-08 |
| ADGRG2 | 1.99 (1.92 - 2.07) | 8.55E-135 | 8.55E-135 | 0.74 (0.67 - 0.81) | 1.45E-09 |
| APLP1 | 1.68 (1.62 - 1.75) | 4.15E-45 | 4.15E-45 | 0.87 (0.83 - 0.92) | 7.06E-07 |
| BOC | 1.62 (1.57 - 1.69) | 5.42E-02 | 5.42E-02 |  |  |
| CCL20***** | 1.49 (1.44 - 1.53) | 2.35E-56 | 2.35E-56 | 1.12 (1.09 - 1.15) | 2.11E-15 |
| CD300LG | 0.60 (0.58 - 0.62) | 2.86E-02 | 2.86E-02 | 1.02 (0.95 - 1.09) | 6.15E-01 |
| CDHR2 | 0.44 (0.41 - 0.47) | 1.00E-143 | 1.00E-143 | 1.03 (0.99 - 1.07) | 1.31E-01 |
| CLUL1 | 1.79 (1.70 - 1.87) | 6.84E-29 | 6.84E-29 | 0.92 (0.86 - 0.97) | 4.38E-03 |
| CNTN1 | 1.79 (1.70 - 1.89) | 2.95E-40 | 2.95E-40 | 0.74 (0.67 - 0.82) | 1.28E-09 |
| COL1A1 | 1.35 (1.31 - 1.39) | 3.36E-02 | 3.36E-02 | 1.09 (0.97 - 1.21) | 1.34E-01 |
| CTRC | 1.89 (1.77 - 2.01) | 5.03E-11 | 5.03E-11 | 0.98 (0.94 - 1.02) | 3.67E-01 |
| CTSD | 0.23 (0.20 - 0.27) | 6.65E-126 | 6.65E-126 | 1.22 (1.14 - 1.29) | 6.07E-10 |
| DNER | 1.21 (1.18 - 1.23) | 6.83E-13 | 6.83E-13 | 0.86 (0.77 - 0.95) | 4.33E-03 |
| EGFR | 1.22 (1.19 - 1.25) | 4.16E-79 | 4.16E-79 | 0.50 (0.42 - 0.58) | 5.19E-17 |
| ENTPD5 | 1.56 (1.47 - 1.65) | 4.34E-04 | 4.34E-04 | 1.02 (0.90 - 1.16) | 7.50E-01 |
| Ep-CAM***** | 1.40 (1.34 - 1.46) | 1.41E-11 | 1.41E-11 | 0.99 (0.96 - 1.01) | 3.26E-01 |
| FGF-21***** | 0.70 (0.67 - 0.73) | 4.58E-78 | 4.58E-78 | 1.08 (1.05 - 1.10) | 4.17E-11 |
| FLT3 | 0.69 (0.66 - 0.73) | 2.09E-27 | 2.09E-27 | 0.84 (0.77 - 0.93) | 3.59E-04 |
| GALNT7 | 0.55 (0.50 - 0.60) | 2.44E-07 | 2.44E-07 | 0.88 (0.77 - 1.00) | 5.19E-02 |
| GDF-15***** | 1.22 (1.18 - 1.26) | 0.00E+00 | 0.00E+00 | 1.62 (1.54 - 1.70) | 1.02E-84 |
| GPA33***** | 0.72 (0.68 - 0.77) | 8.19E-11 | 8.19E-11 | 0.99 (0.97 - 1.01) | 3.32E-01 |
| IL8 | 0.80 (0.77 - 0.83) | 1.63E-32 | 1.63E-32 | 1.08 (1.04 - 1.12) | 5.25E-05 |
| INHBC***** | 0.59 (0.53 - 0.65) | 3.94E-87 | 3.94E-87 | 1.27 (1.18 - 1.36) | 1.82E-11 |
| LPL | 0.69 (0.63 - 0.77) | 2.16E-52 | 2.16E-52 | 0.97 (0.91 - 1.02) | 2.47E-01 |
| MB***** | 0.82 (0.77 - 0.87) | 4.86E-146 | 4.86E-146 | 1.16 (1.10 - 1.21) | 1.13E-09 |
| MFGE8 | 0.91 (0.88 - 0.93) | 3.47E-54 | 3.47E-54 | 1.20 (1.13 - 1.27) | 3.15E-09 |
| MLN | 0.87 (0.83 - 0.90) | 7.27E-95 | 7.27E-95 | 1.10 (1.07 - 1.13) | 1.08E-09 |
| MMP12***** | 0.87 (0.83 - 0.91) | 1.40E-260 | 1.40E-260 | 1.48 (1.42 - 1.55) | 3.74E-65 |
| OMD | 0.94 (0.92 - 0.96) | 1.18E-11 | 1.18E-11 | 0.92 (0.86 - 0.98) | 6.43E-03 |
| OMG | 0.82 (0.76 - 0.88) | 7.17E-29 | 7.17E-29 | 0.90 (0.86 - 0.93) | 9.71E-08 |
| PON3 | 0.71 (0.63 - 0.81) | 6.53E-138 | 6.53E-138 | 0.77 (0.72 - 0.83) | 2.99E-12 |
| SCF | 0.80 (0.73 - 0.88) | 3.89E-08 | 3.89E-08 | 0.85 (0.79 - 0.91) | 5.64E-06 |
| SPINK1***** | 1.20 (1.10 - 1.30) | 8.73E-108 | 8.73E-108 | 1.45 (1.37 - 1.53) | 2.70E-37 |
| TRANCE***** | 0.80 (0.70 - 0.90) | 3.52E-11 | 3.52E-11 | 0.96 (0.91 - 1.00) | 4.95E-02 |
| TWEAK | 0.93 (0.86 - 0.99) | 2.32E-06 | 2.32E-06 | 0.95 (0.86 - 1.04) | 2.45E-01 |
| uPA***** | 0.89 (0.80 - 0.99) | 3.17E-05 | 3.17E-05 | 1.12 (1.02 - 1.22) | 1.28E-02 |
| VMO1***** | 0.89 (0.80 - 1.00) | 9.23E-54 | 9.23E-54 | 1.15 (1.11 - 1.20) | 2.34E-11 |

* Proportional Hazards Assumption violated, see Supplemental Table 16

**Supplemental Table 16: Association between protein levels and time to incident CAD in UK Biobank time-stratified models**

| **Protein** | **0-5 years HR (95% CI)** | **0-5 years P Value** | **0-5 years FDR** | **5-10 years HR (95% CI)** | **5-10 years P Value** | **5-10 years FDR** | **10+ years HR (95% CI)** | **10+ years P Value** | **10+ years FDR** |
| --- | --- | --- | --- | --- | --- | --- | --- | --- | --- |
| ACE2 | 1.82 (1.7 - 1.94) | 5.3E-71 | 7.7E-01 | 1.63 (1.53 - 1.74) | 1.1E-51 | 2.7E-01 | 1.6 (1.49 - 1.72) | 2.4E-36 | 7.8E-01 |
| CCL20 | 1.3 (1.25 - 1.35) | 1.7E-35 | 8.8E-01 | 1.22 (1.18 - 1.27) | 2.9E-23 | 5.7E-02 | 1.13 (1.08 - 1.19) | 1.4E-06 | 7.0E-01 |
| Ep-CAM | 0.86 (0.82 - 0.9) | 2.0E-09 | 1.2E-01 | 0.9 (0.86 - 0.94) | 3.1E-06 | 6.2E-01 | 0.98 (0.93 - 1.03) | 3.8E-01 | 2.2E-01 |
| FGF-21 | 1.26 (1.22 - 1.31) | 2.9E-40 | 8.4E-01 | 1.22 (1.18 - 1.26) | 1.5E-33 | 4.3E-01 | 1.14 (1.1 - 1.18) | 9.1E-12 | 2.8E-01 |
| GDF-15 | 2.17 (2.07 - 2.27) | 5.2E-235 | 2.5E-01 | 2.04 (1.94 - 2.13) | 9.4E-199 | 7.2E-01 | 1.92 (1.81 - 2.03) | 2.4E-106 | 2.7E-01 |
| GPA33 | 0.9 (0.86 - 0.93) | 9.5E-10 | 4.9E-01 | 0.93 (0.91 - 0.97) | 3.7E-05 | 6.6E-01 | 0.98 (0.95 - 1.02) | 3.8E-01 | 1.4E-01 |
| INHBC | 2.1 (1.88 - 2.35) | 2.6E-38 | 2.8E-01 | 1.86 (1.68 - 2.06) | 2.1E-33 | 8.0E-01 | 1.71 (1.53 - 1.92) | 3.5E-20 | 3.2E-01 |
| MB | 1.64 (1.54 - 1.75) | 1.9E-51 | 1.8E-01 | 1.68 (1.59 - 1.78) | 3.5E-68 | 2.9E-01 | 1.53 (1.42 - 1.64) | 9.7E-32 | 4.2E-01 |
| MMP12 | 2.26 (2.12 - 2.41) | 2.5E-130 | 5.9E-01 | 1.96 (1.84 - 2.09) | 2.6E-96 | 8.7E-01 | 1.75 (1.62 - 1.88) | 4.4E-47 | 6.1E-01 |
| SPINK1 | 1.98 (1.82 - 2.16) | 6.2E-55 | 5.0E-01 | 1.68 (1.54 - 1.83) | 4.1E-32 | 4.7E-01 | 1.72 (1.56 - 1.9) | 1.7E-27 | 1.3E-01 |
| TRANCE | 0.82 (0.76 - 0.88) | 1.4E-07 | 7.4E-01 | 0.87 (0.81 - 0.93) | 6.7E-05 | 1.1E-01 | 0.92 (0.85 - 0.99) | 3.1E-02 | 6.4E-01 |
| uPA | 1.38 (1.2 - 1.58) | 4.9E-06 | 3.7E-01 | 1.22 (1.06 - 1.39) | 4.6E-03 | 9.3E-01 | 0.98 (0.83 - 1.16) | 8.3E-01 | 3.1E-01 |
| VMO1 | 1.51 (1.4 - 1.63) | 1.3E-26 | 7.6E-01 | 1.39 (1.3 - 1.49) | 1.0E-20 | 8.2E-01 | 1.31 (1.21 - 1.41) | 2.4E-11 | 6.3E-01 |

**Supplemental Table 17: PROMISE validation protein association with HRP**

| **Protein** | **Univariable Odds Ratio (95% CI)** | **Univariable P Value** | **Univariable FDR Adjusted P Value** | **Multivariable Odds Ratio (95% CI)** | **Multivariable P Value** | **Multivariable FDR Adjusted P Value** |
| --- | --- | --- | --- | --- | --- | --- |
| CGB3 | 0.78 (0.690 - 0.89) | 1.5E-4 | 2.9E-2 | 0.99 (0.82 - 1.19) | 8.9E-1 | 9.7E-1 |
| LEP | 0.80 (0.71 - 0.90) | 1.3E-4 | 2.9E-2 | 0.99 (0.83 - 1.20) | 9.1E-1 | 9.7E-1 |
| PSPN | 1.28 (1.15 - 1.42) | 3.1E-6 | 1.8E-3 | 1.16 (1.00 - 1.35) | 5.0E-2 | 8.6E-1 |
| VAT1 | 0.36 (0.20 - 0.62) | 3.1E-4 | 4.4E-2 | 0.42 (0.23 – 0.77) | 5.2E-3 | 8.6E-1 |
